# Therapeutic Promise of Mitophagy in Cancer: Advancing from Small-Molecule Regulation to Nanotechnology-Enhanced Targeting Therapy

**DOI:** 10.7150/thno.129867

**Published:** 2026-01-30

**Authors:** Ping Chen, Guohao Liu, Jiani Yin, Ling Sun, Xiaoming Wang, Bing Wang, Qiyong Gong, Kui Luo

**Affiliations:** 1 Department of Radiology, Institution of Radiology and Medical Imaging, Huaxi MR Research Center (HMRRC), Frontiers Science Center for Disease-Related Molecular Network, National Clinical Research Center for Geriatrics, State Key Laboratory of Biotherapy, West China Hospital, Sichuan University, Chengdu 610041, China.; 2 Psychoradiology Key Laboratory of Sichuan Province, West China Hospital, Sichuan University, Research Unit of Psychoradiology, Chinese Academy of Medical Sciences, Chengdu 610041, China.; 3 Xiamen Key Lab of Psychoradiology and Neuromodulation, Department of Radiology, West China Xiamen Hospital of Sichuan University, Xiamen 361021, China.; 4 XiangYa School of Medicine, Central South University, 172 Tongzipo Road, YueLu District, 410013 Changsha, Hunan, China.

**Keywords:** mitophagy, cancer therapy, small molecule regulators, nanotherapeutics

## Abstract

Mitophagy, a selective autophagic pathway that clears damaged or dysfunctional mitochondria, has emerged as a promising therapeutic approach. Mitophagy maintains a delicate balance between cell survival and death, while mounting evidence suggests that it predominantly promotes tumor cell survival under stress, particularly in responses to cancer therapy. Moreover, aberrant regulation of mitophagy results in cancer pathology with characteristic hallmarks, including remodeling of metabolic plasticity, maintenance of cancer stem cell characteristics, and immune regulation of the tumor microenvironment. This review synthesizes multifaceted roles of mitophagy in cancer biology, from tumor initiation and progression to therapy responses. It also summarizes molecular mechanisms underlying mitophagy. How cancer cells exploit mitophagy to survive therapy has been harnessed to develop therapeutic strategies. We elaborate the evolution of mitophagic therapy from small-molecule modulators to nanotechnology-based targeted delivery systems. Finally, we highlight the promise of targeting mitophagy in overcoming treatment resistance and improving clinical outcomes for patients.

## 1. Introduction

Mitochondria are frequently called the “powerhouse” of the cell, producing adenosine triphosphate (ATP) via oxidative phosphorylation. This process is the basis for controlling cellular energy metabolism and supporting a host of fundamental life functions. Concomitantly, mitochondria are the major site of reactive oxygen species (ROS) generation, which are highly implicating in physiological processes including apoptosis and the regulation of calcium homeostasis 1,2. Cooperative control of these physiological events is essential to preserve cell homeostasis. However, it should be stressed that mitochondrial function is very vulnerable to environmental stress. Situation of hypoxia, deficiency of nutrients, and damage from oxidative stress can result in dysfunctional including reduction in mitochondrial membrane potential, excess release of ROS and even impairment to mitochondrial DNA (mtDNA) 3. If the damaged mitochondria are not in time removed, they will not only cause the disturbance of cellular energy homeostasis, but can also lead to DNA mutation via ROS accumulation and then promote malignant transformation or accelerate progression of age-related diseases 4,5. This succession of events underscores the importance of the quality control mechanisms of mitochondria. Therefore, mitochondrial quality control (MQC) mechanisms are irreplaceable in preserving intracellular homeostasis 6,7. As a key player in MQC, mitophagy as a hot topic is booming at the crosscut of life sciences and clinical medicine 8,9.

Mitophagy is an evolutionarily well-conserved form of selective autophagy that selectively recognizes and removes damaged mitochondria, resulting in organelle turnover as well as the recycling of their raw materials 10. This process is widely present in various model organisms, including yeast, nematodes, fruit flies, and mammals, and mainly involves three core steps: (1) mitochondrial depolarization, i.e., loss of membrane potential in damaged mitochondria; (2) formation of a pre-autophagosome (a double-membrane structure) that engulfs the damaged mitochondria, forming a mitophagosome; (3) fusion of the mitophagosome with a lysosome, ultimately completing the degradation and recycling of mitochondrial components 5. As research advances, three principal molecular mechanisms of mitophagy have been revealed. The ubiquitin (Ub)-dependent pathway, via the PTEN-induced putative kinase 1 (PINK1)-Parkin axis, identifies damaged mitochondria through cascading ubiquitination and recruits autophagy receptors, such as optineurin (OPTN) and sequestosome-1 (p62/SQSTM1, selective autophagy receptor that mediates the association of ubiquitylated mitochondria with autophagosomes), to mediate clearance; the receptor-mediated pathway relies on intrinsic outer mitochondrial membrane (OMM) receptors (e.g., BCL2-interacting protein 3 (BNIP3), BCL2-interacting protein 3-like (BNIP3L)/NIX, FUN14 domain-containing 1 (FUNDC1)), these receptors are directly connected to autophagic membranes via their light chain 3 (LC3)-interacting regions (LIRs), and the activity is modulated by post-translational modifications such as phosphorylation 11, 12; and mitochondria-derived vesicle (MDV, a vesicular structure for selective clearance of mitochondrial components)-mediated mitophagy serves as a complementary mechanism, and it allows precise clearance of damaged mitochondrial components independent of classical autophagic factors including autophagy-related protein 5 (ATG5) and microtubule-associated protein 1 LC3. Collectively, these pathways coordinate and assign tasks to ensure selective clearance of mitochondria under varying levels of damage in different cellular environments 13, 14.

The physiological significance of mitophagy extends far beyond simple organelle quality control. Physiologically, the removal of senescent mitochondria requires mitophagy, involving the maintenance of stemness of stem cells, and control of mitochondrial-clearance (driven by erythrocyte maturation), but also offering adaptive regulatory support for cardiomyocytes adapting to hypoxic environments 15,16. The completion of these physiological functions underscores the essentiality of mitophagy in the regulation of cellular homeostasis. Nevertheless, under pathological conditions, mitophagy disorder is directly correlated with the occurrence and deterioration of multiple diseases 17,18. In particular, mitophagy dysfunction can lead to neurodegenerative diseases such as Parkinson's disease, where the PINK1 or Parkin mutation is responsible for defective mitophagy and dopaminergic neuronal apoptosis 19, and metabolic syndrome with aggravating insulin resistance through dysregulated mitophagy resulting in the acceleration of fatty liver disease 20. While detailed mechanistic gain of insights during mitophagy dysregulation is disparate among these diseases, it clearly indicates the evidence underpinning its centrality as a regulatory hub in disease pathogenesis. Notably, it should be underlined that mitophagy plays a prominent double-edged sword role in cancer development: while at the phase of early tumorigenesis, mitophagy is anti-tumorigenic by clearing ROS-damaged mitochondria and maintaining genome stability 21, whereas at late stage of tumor progression, it enables tumor cells to adjust to hypoxic microenvironmental conditions, escape immune surveillance and resist chemotherapeutic drugs to ultimately support cell survival/growth 22. Such differential functional change of mitophagy throughout different tumor stages characterizes the function of mitophagy in cancer pathology. Its dual role implies a possibility that selective modulating mitophagy might be an important innovation in exploring new ways of cancer treatment.

In the last years progress in mitophagy research has been exceptional 23,24. These progresses offer a sound theoretical basis for the design of mitophagy-targeted therapeutic approaches. Small-molecule modulators, as important regulation tools, could selectively regulate the downstream key factors of mitophagy including PINK1/Parkin, autophagic receptor and lysosome, to activate the initial step of autophagy or block it 20. They are tools essential for mitophagy modulation thanks to high specificity and well-described modes of action these compounds play an important role in the field of mitophagic activity. More importantly, tannin I decreases mitophagy activity through the inhibition of PINK1-Parkin pathway, further resulting in apoptosis of hepatocellular carcinoma (HCC) cells; while, ONX0912 increases mitochondrial recycling efficiency by the activation of this pathway, to realize cell death 25,26. These two small molecules target towards mitophagy pathway through distinct mechanisms, they are in a complementary manner to facilitate the regulatory diversity and practicability of this process. However, small molecule drugs have limited therapeutic effects due to poor target specificity and high systemic toxicity, thus hindering their clinical application. The emergence of nanotechnology has provided a new direction for solving these problems 27: through surface modification, mitochondria-targeted delivery systems constructed from nanocarriers such as liposomes and polymers can be enriched in the mitochondria of tumor cells 28, significantly improving drug delivery efficiency and regulatory precision, and have shown synergistic anti-tumor effects in animal models of lung cancer, breast cancer, and liver cancer. Based on this, this review systematically summarizes the molecular mechanisms of mitophagy, its significance in cancer treatment, and targeted regulatory strategies, aiming to provide a comprehensive reference for basic research in this field and lay a theoretical foundation for future clinical translation.

## 2. Molecular Mechanisms of Mitophagy

Mitophagy is a form of selective autophagy named in 2005. Under physiological conditions, it maintains mitochondrial and cellular homeostasis by clearing damaged or dysfunctional mitochondria, and is a key lysosomal degradation pathway 11,29. Since its discovery, its core molecular mechanisms and roles in health and disease have been gradually revealed. Because the initiation of mitophagy (characterized by the encapsulation of damaged mitochondria by autophagosomes) is a potential therapeutic target for mitophagy intervention, this process has also become a research hotspot 30,31: it is driven by a variety of autophagy adaptor proteins or receptors and can respond to a series of cellular and mitochondrial stimuli ranging from mitochondrial damage to metabolic reprogramming 32. In addition to the initiation mechanism, mitophagy is also strictly regulated by complex signaling pathways and molecular networks to ensure that damaged mitochondria are precisely and selectively degraded 33. Mechanistically, mitophagy is mainly divided into three types: Ub-dependent, receptor-dependent, and MDV-mediated.

### 2.1. Ubiquitin-Dependent Mitophagy

**Figure 1:**
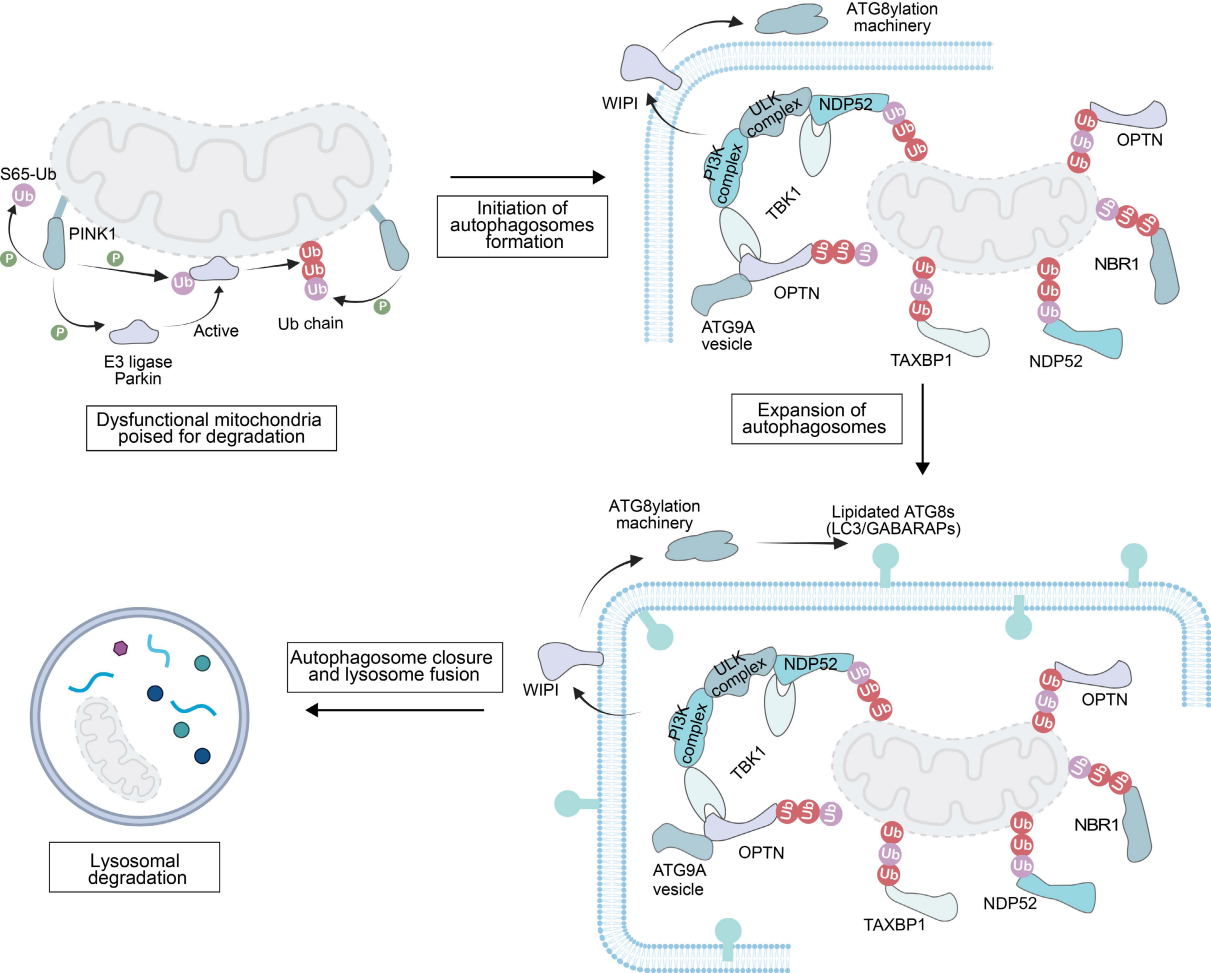
Mechanistic illustration of mitophagy through the ubiquitin-dependent pathway. Created in https://BioRender.com.

The degradation of damaged mitochondria is initiated by a series of strictly regulated ubiquitination processes (**Figure 1**). First, PINK1 kinase phosphorylates Ub, a modification that mediates the recruitment of the E3 Ub ligase Parkin to mitochondria 34. Subsequently, Parkin ubiquitinates OMM proteins, forming a positive feedback loop to amplify the degradation signal 35.

These phosphorylated Ub chains then serve the role of “molecular signal tags”, recruiting autophagy receptor proteins such as nuclear dot protein 52 (NDP52), OPTN and human T-cell leukemia virus type I binding protein 1 (TAX1BP1) 36. On these damaged mitochondria, the receptor can also recruit additional autophagy-related cellular machinery (ATG9A vesicles, the phosphatidylinositol 3-kinase complex and Unc-51-like kinase‌ (ULK) complex). These factors are involved in the initiation of autophagosome precursors *de novo* synthesis at the surface of damaged mitochondria 37,38.

Meanwhile, PI3P-binding proteins including the WD-repeat protein and phosphoinositide-interacting (WIPI) family mobilize the ATG8 conjugation system by binding to PI3P. This system mediates the anchoring of autophagy-related protein 8 (ATG8) to phosphatidylethanolamine on the autophagosome precursor membrane 39. The membrane-anchored ATG8s not only support the expansion of the autophagosome precursor but also assist in recruiting more autophagy adaptor proteins 40. Finally, the autophagosome precursor continuously extends and closes to form a complete autophagosome, which is then transported to the lysosome, completing the final degradation of the damaged mitochondria.

#### 2.1.1. The PINK1-Parkin pathway

In damage-induced mitophagy, the core mechanism is the ubiquitination modification of OMM proteins and this modification provides a targeting signal for the selective clearance of dysfunctional mitochondria. The serine/threonine kinase PINK1 and the E3 Ub ligase Parkin (an RBR family E3 Ub protein ligase) are key regulatory factors in this pathway (**Figure 1**). PINK1, encoded by the Parkinson's disease-associated gene PARK6, is a crucial serine/threonine kinase in MQC process 41. The function of its full-length protein (containing 581 amino acids) depends on the synergistic action of multiple domains 42: the N-terminal mitochondrial/matrix targeting sequence (MTS) and transmembrane domain (TM) are responsible for mediating mitochondrial targeting and localization; the core kinase domain contains three insertion loops (Ins1, Ins2, Ins3), with Ins3 acting as a substrate-binding loop and being a key structure for PINK1 phosphorylation of Ub and Parkin; and the C-terminal extension region (CTE) is responsible for maintaining protein structural stability 43,44. After flanking the kinase domain, the N-terminal extension (NTE) and CTE are indispensable for binding to translocase of OMM (TOM) 20 subunit in the translocase of the outer membrane translocase complex, thereby facilitating PINK1 activation, while an N-terminal TOM70 interaction region (TIR) enhances activation by binding to TOM70 44-47. *In vitro* studies have shown that endogenous PINK1 is continuously synthesized in the cytosol as a ~63-68 kDa precursor, ​which is critical for its subsequent mitochondrial import and activation 48. In healthy polarized mitochondria, TOM20 recognizes the MTS, and directs PINK1 to the TOM40 translocation pore with assistance from TOM22 and TOM70, ultimately delivering PINK1 to the TIM (translocase of the inner mitochondrial membrane (IMM)) 23 complex on the IMM 49. Here, PINK1 is cleaved by mitochondrial processing peptidase and presenilin-associated rhomboid protease to generate a 52 kDa isoform, which is rapidly degraded via the ubiquitin-proteasome system. As a result, PINK1 is typically undetectable in cells with normally polarized mitochondria 42. In contrast, in damaged or depolarized mitochondria, the adenine nucleotide translocator (ANT) complex interacts with TIM44 to inhibit PINK1 translocation to TIM23, preventing its cleavage by presenilin associated rhomboid like (PARL) 50. Meanwhile, the CTE of PINK1 binds to TOM7 to stabilize PINK1 on the OMM 51, 52. With assistance from the TOM complex, PINK1 undergoes trans-autophosphorylation, inducing a conformational change to destabilize the PINK1 dimers. The Ub chains on OMM proteins at serine 65 (S65) are phosphorylated to create a signal for Parkin recruitment 53. Since PINK1 functions as both a Parkin kinase and a monoubiquitin kinase, the accumulated phosphorylated Ub enhances Parkin affinity for mitochondria, ​thereby initiating mitophagy 54.

Parkin, encoded by the parkinson disease 2 (PARK2) gene, is a RING-between-RING (RBR) E3 Ub ligase. Its structure comprises several zinc-coordinating domains: ubiquitin-like (UBL), RING1, RING2, in-between ring fingers, and RING0 55-57. In its basal state, Parkin retains an autoinhibited conformation because the UBL domain and the repressor element of Parkin (REP) cooperatively block the E2 binding site on RING1, while the RING0 domain masks the catalytic cysteine (Cys431) in RING2 58. Upon PINK1-mediated phosphorylation, Parkin undergoes conformational rearrangement. First, phosphorylated S65 Ub binds to Parkin to displace the inhibitory UBL domain and extend REP to disrupt autoinhibition 59. Next, the UBL domain is phosphorylated by PINK1, promoting its binding to a basic region on RING0, which triggers a conformational change of RING2 and Cys431 exposure for Ub thioester intermediate formation 60. Through these changes, Parkin transitions to an active E3 ligase. OMM proteins, including mitofusins (MFN1/2), mitochondrial Rho GTPase 1 (Miro1), and voltage-dependent anion channel 1 (VDAC1), are ubiquitinated by Parkin. Concurrently, these substrates are phosphorylated by PINK1 to amplify Ub chains and enhance Parkin recruitment. Additionally, intermolecular interactions between phosphorylated UBL and RING0 boost the activation efficiency, promoting Parkin aggregation at damage sites for efficient mitochondrial clearance 43. The core abnormal pattern of the PINK1-Parkin pathway is selective activation or inactivation of the pathway in tumor cells. Inactivation of the pathway in early stages of cancer leads to ROS accumulation and tumorigenesis promotion, while activation of the pathway in late stages of cancer enhances tumor drug resistance and becomes a key node for targeted therapy 42. Upregulation of the PINK1/Parkin expression facilitate clearance of both normal and abnormal mitochondria in tumor cells, and tumor cells shift their metabolic preference towards glycolysis for energy supply for their adaptation to a hypoxic tumor microenvironment (TME) 61. It can also promote survival and chemoresistance of tumor cell by maintaining mitochondrial homeostasis and reducing ROS levels.

#### 2.1.2. Other E3 ligases in mitophagy

Beyond Parkin, several other E3 ligases regulate mitophagy, ​and they are classified into RING-type, HECT-type, and RBR-type based on their structures and Ub transfer mechanisms 62. Ubiquitin-conjugating enzyme E2 binding protein 1 (ARIH1), an RBR family member with structural similarity to Parkin but with distinct expression patterns in cancer cell lines and pluripotent stem cells, catalyzes polyubiquitination of damaged mitochondria in tumor cells. This process directs damaged mitochondria to autophagy, protecting tumor cells against chemotherapy-induced cell death and potentially contributing to drug resistance 63. Seven in absentia homolog 1 (SIAH1), a RING-type ligase, functions via the PINK1-Synphilin-1-SIAH1 complex. After PINK1 binds to Synphilin-1, SIAH1 is recruited to ubiquitinate mitochondrial proteins independently of Parkin, which triggers LC3 and Lamp1 recruitment to initiate mitophagy 19. Notably, SIAH1 recruitment does not require PINK1 kinase activity, distinguishing from the canonical pathway 64. Mitochondrial E3 Ub protein ligase 1 (MUL1), an OMM-localized ligase with a cytosolic RING domain, shares substrates with Parkin (e.g., dynamin-related protein 1 (Drp1), MFN) and exhibits both Ub ligase activity (promoting MFN degradation) and small ubiquitin-like modifier (SUMO) ligase activity (stabilizing Drp1) 65. The resulted Ub chains recruit autophagy adaptors (OPTN, NDP52, p62) via their LIRs to tether mitochondria to autophagosomes. Furthermore, MUL1 compensates for the PINK1/Parkin deficiency and functions independently of PINK1 66.

#### 2.1.3. Mitophagy receptors

In the PINK1-Parkin pathway, Ub chains on mitochondria recruit autophagy receptors via ubiquitin-binding domains (UBDs) and LIRs. These receptors bridge damaged mitochondria to ATG8-family proteins on phagophores, facilitating autophagosomal encapsulation and subsequent lysosomal degradation. ​Specifically, mitophagy receptors, which are recruited to the OMM only after Parkin activation and ubiquitination, feature a dual-domain architecture: an N-terminal UBD and a C-terminal LIR. This structure enables them to bind to mitochondrial Ub via the UBD and autophagosomal LC3/GABARAP via the LIR 67. ​The majority of these receptors belong to the SQSTM-1-like receptor (SLR) family, which include p62/SQSTM1, neighbor of BRCA1 gene 1 (‌NBR1), calcium binding and coiled-coil domain 1, NDP52, TAX1BP1, and OPTN 68, 69.

p62/SQSTM1, a multimeric modular protein, is the first identified selective autophagy receptor 70. It utilizes its N-terminal Phox and Bemlp (PB1) domain to mediate homo/heterodimerization 71-74. It also acts as a molecular bridge between linking ubiquitinated cargos and phagophores, driving phase-separation of p62 bodies and the formation of the scaffold autophagosome assembly 75. This process is elegantly regulated. Phosphorylation at S407/S403 enhances the interaction between the Ub activating (UBA) enzyme domain of p62 and Ub, while ubiquitination at K420 helps recruit the Kelch-like ECH-associated protein 1 (Keap1) - Cullin-3 complex to stabilize p62 bodies and disrupt UBA homodimerization. In addition, oligomerization of p62 is critical for efficient substrate sequestration 76. Notably, p62 mediates mitochondrial ubiquitination independently of the PINK1-Parkin pathway. For example, in Dnm1l-knockout murine tissues, mitophagy proceeds without Parkin, while sqstm1 ablation (loss of p62) significantly reduces the degree of mitochondrial ubiquitination. Mechanistically, SQSTM1 recruits the E3 ligases Keap1 and RING-box protein 1 (RBX1), subsequently forming a Cullin-RING ligase complex that directly promotes the ubiquitination of mitochondrial proteins 73.

NBR1 interacts with the PB1 domain of p62 through its own PB1 domain, but due to the characteristics of its PB1 domain, NBR1 always exists in a monomeric form 77,78. Unlike p62, the PB1 domain of NBR1 can bind to other PB1 domain-containing proteins, either integrating into p62 polymers or "capping" and regulating p62 polymers. Specifically, the additional coiled-coil (CC) domain of NBR1 promotes its own dimerization; the ubiquitin-binding domain (UBA) is responsible for binding ubiquitinated substrates; the LC3-interacting region (LIR) motif interacts with ATG8 family proteins; the amphipathic helix (AH) domain targets lysosome-associated membrane protein 2 (LAMP2)-positive endosomes and peroxisomes; and the FW domain mediates diverse intermolecular interactions—the synergistic action of these domains collectively highlights the substrate specificity of NBR1 77,79.

NDP52 and OPTN are rapidly recruited to damaged mitochondria through their ubiquitin-binding domains (UBDs) and subsequently phosphorylated by TANK-binding kinase 1 (TBK1, a key kinase that regulates mitophagy receptor activity) 80. Notably, this phosphorylation not only enhances their retention within mitochondria but also increases their binding efficiency to Ub 81-83. NDP52 has the following domains: An N-terminal SKIP carboxyl-homology (SKICH) domain, a central coiled-coil region, a C-terminal Zn finger (ZF) that binds to Ub and an atypical C-terminal LIR motif, that adopts its interaction with LC3/GABARAP family members 84,85. TBK1 can phosphorylate OPTN at serine (Ser) 473/513 and Ser177, which increases its affinity for Ub and ATG8 family proteins 86,87. Importantly, phosphorylation alteration of its LIR motif improves OPTN binding affinity to lipidated LC3, thereby facilitating its isolation membrane recruitment. In addition to the conventional LIR-dependent interaction, NDP52's SKICH domain can instead function in an alternative way by recruiting retinoblastoma 1-induced coiled-coil 1 (RB1CC1) towards the ATG14/ATG16L complex; meanwhile OPTN's leucine zipper domains are able to mediate binding with ATG9A-positive vesicles 88. These interaction axes are important in mitophagy, and their disruption greatly compromises mitophagy by affecting cargo receptors and autophagosome nucleation scaffolds at the same time 89.

TAX1BP1, which is a homologue of NDP52, is expressed at relatively high and broader levels in various kidney and brain tissues. Functionally, TAX1BP1 is more than a typical LIR-containing protein, as it harbors not only the canonical LIR motif but also a non-canonical LIR (Leu-Val-Val) for binding to LC3C and executing substrate engulfment into autophagic precursors. Importantly, mutations in the LIR motif directly disrupt the localization of TAX1BP1 on autophagosomes 90.

Moreover, these mitophagy receptors are recruited to damaged mitochondria to initiate downstream mechanisms of autophagosome biogenesis. Upon activation, the ULK complex, comprising ATG13, FIP200, ATG101 and either Unc-51-like autophagy-activating kinase 1 (ULK1, a central regulator of autophagy initiation) or ULK2, can directly phosphorylate multiple proteins, including ATG4B, ATG9, subunits of PI3K complex I (ATG14L, BECN1), and autophagy and beclin-1 regulator (AMBRA) 1 91. Concurrently, ATG9 vesicles from the trans-Golgi apparatus are localized close to the autophagosome formation site during the early stages of autophagy 92. After recruitment of ATG9 vesicles, the PI3K complex I is also recruited to these sites, where the phosphatidylinositol 3-phosphate (PtdIns3P) complex is generated 93. This complex regulates autophagy by recruiting various Ptdlns3P-binding proteins such as B-cell lymphoma 2 (BCL-2), Bax-interacting factor 1 (BIF-1), and AMBRA-1. Next, the ATG16L1 complex interacts with WIPI2b through its ATG16L1 component and relocates to the pre-autophagosome membrane (an isolation membrane) in response to Ptdlns3P 94. Subsequently, the ATG16L1 complex interacts with ATG3, promoting covalent attachment of lipid groups to Atg8-family proteins 95. Here, ATG7, ATG3, and ATG16L1 complexes act as E1, E2, and E3 enzymes, respectively, and they catalyze covalent binding of ATG8-family proteins to phosphatidylethanolamine on the autophagosome precursor/isolation membrane, thereby driving membrane expansion through coordinated mechanisms 96.

### 2.2. Receptor-Mediated Mitophagy

**Figure 2:**
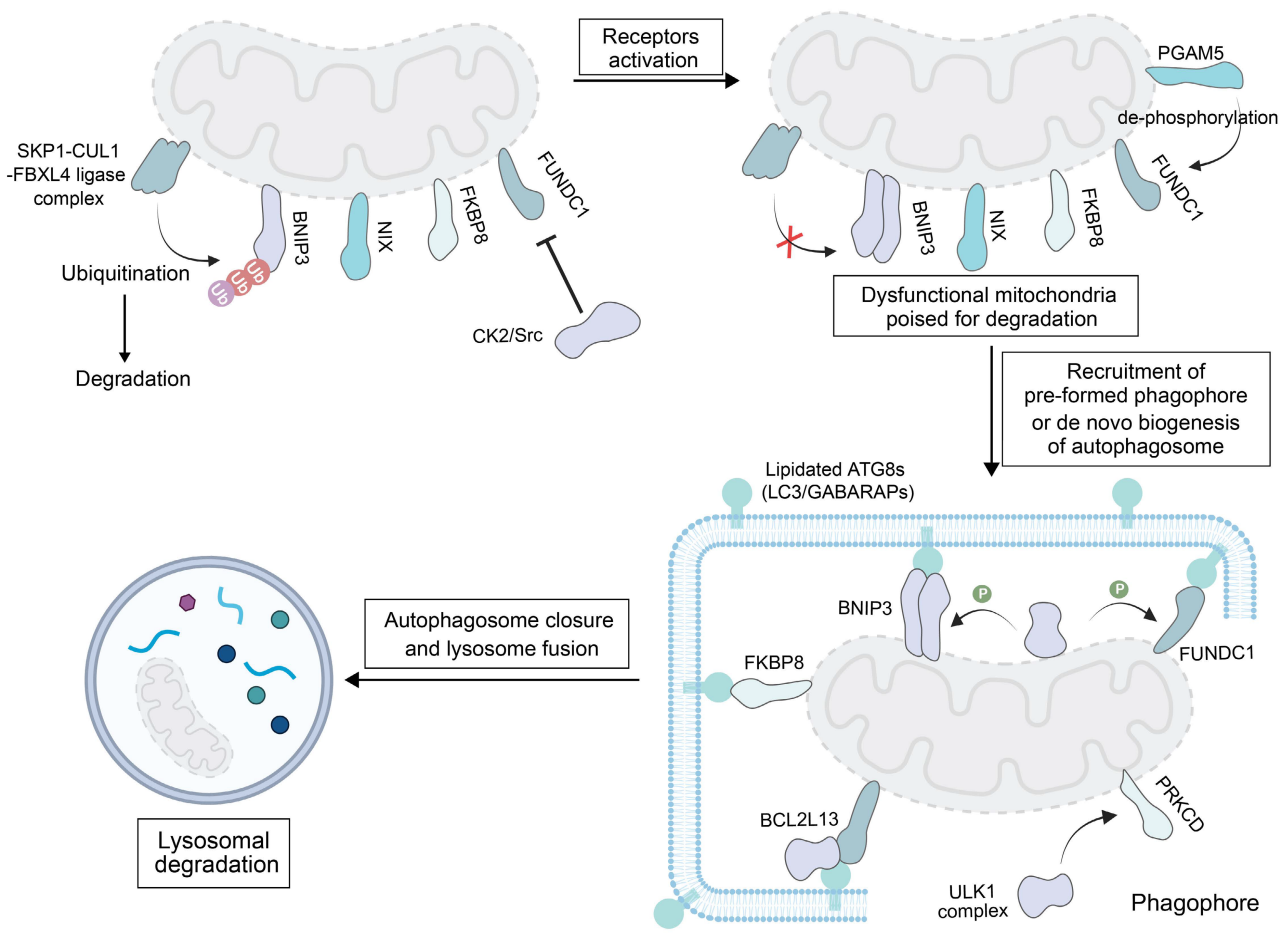
Mechanistic illustration of mitophagy through ubiquitin-independent pathways. Created in https://BioRender.com.

Mitophagy can also proceed via a ubiquitin-unrelated pathway, which relies on specific receptor proteins located on the OMM. Identified receptors mediating this process include BNIP3, BNIP3L/NIX, FUNDC1, BCL2 like 13 (BCL2L13) and FK506-binding protein 8 (FKBP8) 21 (**Figure 2**). This pathway is only activated under specific stress conditions to assist the PINK1- Parkin pathway in normal cells. Prior to mitophagy initiation, these receptors remain inactive on the OMM and their activation is triggered through coordinated processes, such as dimerization modulation, phosphorylation, and dephosphorylation events 97. When activated, they can use their LIR motif to bind to ATG8-family proteins, and damaged mitochondria are directed to pre-formed phagophores or *de novo* formation of autophagosomes is triggered on the mitochondria.

BNIP3 and NIX are mammalian mitophagy receptors which share 65% sequence identity 98. Under basal conditions, BNIP3 displays an inactive cytosolic monomer. The low levels of BNIP3 and NIX are maintained by ubiquitination and proteasomal degradation via the S-phase kinase-associated protein 1 (SKP1)-CUL1-FBXL4 (F-box/LRR-repeat Protein 4) Ub ligase complex that are localized on the outer membrane 99. ​Upon exposure to hypoxic stress, BNIP3 forms homodimers via a glycine zipper motif in its transmembrane domain to anchor onto the OMM 100. Phosphorylation of BNIP3 on S17 by ULK1 activates mitophagy and stabilizes BNIP3. Meanwhile, c-Jun N-terminal kinase 1/2 (JNK1/2, a kinase activating BNIP3/NIX-mediated mitophagy) and protein phosphatase 1/2A (PP1/2A) modulate phosphorylation of BNIP3 under hypoxia, regulating its stability via the ubiquitin-proteasome pathway, and the activity is ultimately determined by its phosphorylation level relative to the total protein content 101. ​In a similar manner as BNIP3, NIX dimerization is required for interaction with ATG8 family members and induction of mitophagy 102. The dephosphorylation of its Ser212 is crucial for this process, and a Ser212 mutation impairs its homodimer formation and mitophagy induction 103. However, the phosphorylation of serine residues near the LIR motif of NIX (Ser34 and Ser35) enhances its affinity to autophagosomes. Highly expressed NIX can also promote epithelial-mesenchymal transition (EMT) in tumor cells to facilitate tumor metastasis.

FUNDC1, a ubiquitously expressed OMM protein involved in mitochondrial dynamics, is a ubiquitin-independent receptor tightly regulated by reversible phosphorylation. When cells are in a quiescent state, casein kinase 2 (CK2) kinase phosphorylates FUNDC1 at Ser13, while Src kinase modifies the Tyr18 site of this protein. These phosphorylation events block the binding of FUNDC1 to LC3B, thereby inhibiting the receptor from triggering mitophagy under hypoxia or FCCP treatment 104. When mitophagy is stimulated, FUNDC1 becomes activated through two coordinated modifications: phosphoglycerate mutase family member 5 (PGAM5) removes the phosphate group from Ser13 (dephosphorylation), and ULK1 adds a phosphate group to Ser17 (phosphorylation). These changes promote the interaction between FUNDC1 and ATG8 proteins on impaired mitochondria 105.

Studies have revealed that FKBP8 exerts mitophagic activity in cells where both FKBP8 and LC3A are overexpressed 106. In these cells, FKBP8 interacts with LC3A to initiate mitophagy. Additionally, FKBP8 is recognized as a key regulator of mitophagy induced by iron deprivation with DFP (deferiprone) but is independent of BNIP3 and NIX 107.

BCL2L13 is a human counterpart of ATG32, a mitophagy receptor originally identified in yeast. Beyond binding to LC3B via its LIR motif, BCL2L13 has been shown to recruit ULK1 to mitochondria when cells are treated with carbonyl cyanide m-chlorophenylhydrazone (CCCP, a chemical inducer of mitochondrial depolarization and mitophagy) 108. Interestingly, the function of BCL2L13 to drive mitophagy appears to be independent of its role in regulating the mitochondrial shape, because severe mitophagy impairment is observed in a BCL2L13 mutant (with a non-functional LIR motif that cannot bind to LC3B) but the mitochondrial morphology is unaffected 109. At this stage, the specific mechanism of activating BCL2L13 by CCCP treatment remains unclear; however, evidence confirms that mitochondrial damage is a trigger for BCL2L13-mediated mitophagy.

It is worth noting that, in contrast to various transmembrane receptors that initiate autophagy by interacting with FIP200 in the ULK complex, such as FUNDC1, BCL2L13, protein kinase C delta (PRKCD) and the family with sequence similarity 134, member C (FAM134C), NIX and BNIP3 do not interact with FIP200 110. Instead, they directly recruit downstream WIPI proteins to the mitochondrial surface. These WIPI proteins​ interact with the upstream ULK1 complex via the ATG13/101 subunits to initiate mitophagy 111. These findings suggest that the downstream autophagy factors may promote the recruitment of the upstream components, effectively reversing the classical sequence of autophagy initiation events. The WIPI-ATG13 axis may serve as a broadly utilized interface for transmembrane cargo receptors. Additionally, studies have revealed that FKBP8 can bind to WIPI2 and it can also interact with FIP200, suggesting that it may activate mitophagy through both WIPI-dependent and FIP200-dependent pathways 112.

The core pathological feature of receptor-mediated mitophagy in tumor cells is an abnormally high expression level of receptors such as BNIP3 and FUNDC1, which change affect to inhibits hypoxic adaptation of tumor cells by regulating the efficiency of mitochondrial clearance, thus providing specific therapeutic targets for hypoxic tumors. In a hypoxic tumor microenvironment, BINP3 is persistently highly expressed, which mediates the clearance of mitochondria and simultaneously inhibits apoptosis. In addition, FUNDC1 at a high expression level can upregulate the expression of lactate dehydrogenase A, synergizing with the Warburg effect.

### 2.3. MDV-Mediated Mitophagy

MDV, a small vesicular structure that selectively packages mitochondrial cargoes (e.g., proteins damaged by oxidation), which buds off from mitochondria under both basal and oxidative stress conditions 13. MDVs formed in response to oxidative stress are dependent on PINK1 and Parkin; unlike canonical autophagy, these vesicles are degraded by lysosomes without the need to form autophagosomes. In contrast, MDV biogenesis under basal conditions does not require PINK1 or Parkin, but instead relies on proteins involved in mitochondrial fission 113. While both types of MDVs are degraded via the lysosomal pathway, the specific mechanisms underlying their delivery to endolysosomes remain to be revealed, particularly for basal MDVs.

**Figure 3:**
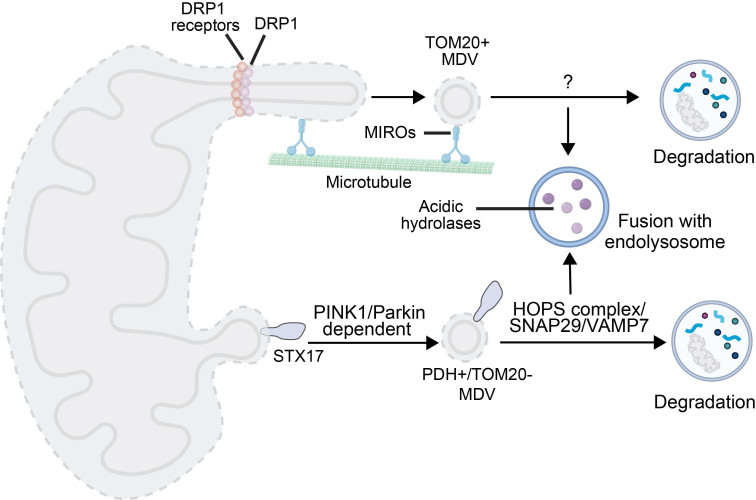
Mechanistic illustration of mitophagy through MDVs. Created in https://BioRender.com.

MDVs have a size of 60-150 nm and they encapsulate OMM, IMM, and matrix components 114. In yeast, MDVs serve as a protective mechanism against stress-induced depolarization, while in mammalian HeLa cells under oxidative stress, they mediate lysosomal clearance of damaged mitochondrial components 113. In contrast to indiscriminate mitophagy that eliminates entire mitochondria, MDVs fine-tune proteome turnover for repair and remodeling, which may lead to the observed tissue- and cell-type-specific differences in protein turnover. Their degradation is independent of mitochondrial depolarization, the presence of core fission proteins, and the abundance of traditional autophagic regulators (e.g., ATG5, LC3). In HCC or breast cancer, MDVs are also often upregulated under hypoxic or chemotherapeutic stress conditions, which enhances invasiveness and chemoresistance of tumor cells. Three specialized routes have been characterized: TOM20-positive, pyruvate dehydrogenase -positive (PDH+), and Mieap-induced vacuole (MIV)-mediated mitophagy (**Figure 3**) 115.

Formation of TOM20-positive MDVs is initiated by Miro1/2-induced mitochondrial protrusions. DRP1 is recruited to form foci for scission, releasing MDVs associated with the TOM complex. Their generation is negatively regulated by Ub specific peptidase 30. The generation process is independent of PINK1/Parkin, while Parkin synergizes with Toll-interacting proteins during lysosomal transport of TOM20-positive MDVs 113, 116.

PDH-positive MDV-mediated degradation occurs in two stages. Under oxidative stress, oxidized matrix proteins trigger PINK1 accumulation, leading to phosphorylation of Ub and recruitment of Parkin to enhance OMM ubiquitination. OMM ubiquitination in synergy with syntaxin 17 (STX17, a protein involved in MDV budding and autophagosome-lysosome fusion) recruitment results in MDV budding 114. Fusion of MDVs with endolysosomes depends on soluble N-ethylmaleimide-sensitive factor attachment protein receptor (SNAREs) (e.g., synaptosome-associated protein 29 (SNAP29), vesicle-associated membrane protein 7 (VAMP7)) and the homotypic fusion and protein sorting (HOPS) complex 117. This process is often triggered by mild injury, and it complements mitophagy in the PINK1/Parkin context. Interestingly, PDH-positive MDVs generation is exploited by autophagy-deficient cancer cells for survival 115.

MIV-mediated mitophagy represents a novel lysosomal degradation pathway for elimination of oxidized mitochondrial proteins 118. This process is triggered when Mieap-induced lysosome-like organelles formation is inhibited, and Mieap is induced to generate vacuole-like structures. As a Mieap-dependent pathway component, the MIV exerts its function by engulfing unhealthy mitochondrial components and mediating their degradation through lysosome accumulation, which is regulated by BNIP3 and NIX. A recent study has revealed that the generation of MIV is also regulated by the p53-Mieap axis. Inactivation of p53 disrupts MIV generation, which in turn leads to the accumulation of unhealthy mitochondria 62-64.

## 3. Mitophagy in Cancer

Mitophagy, a core mechanism of selective autophagy, exerts pronounced bidirectional regulatory effects on tumorigenesis and progression. During early malignant transformation, mitophagy, through selective autophagy mechanisms, clears damaged mitochondria, maintaining genomic stability and thus reducing ROS-mediated DNA damage, playing a crucial tumor-suppressive role. Nonetheless, as tumorigenesis evolves towards more advanced stages, the mitophagy processes make a transition from: suppressor to distributor of tumor growth driving impaired response initiating and execution phase of apoptosis (and cross talk with other cellular homeostatic pathways) adding metabolic flexibility to cancer cells challenged by hypoxia and nutrient poor microenvironments ultimately determining survival of the tumor cell 21, 22. The “double-edged sword” of mitophagy indicates that the modulation and manipulation of mitophagy can be a new strategy to combat drug resistance in cancer therapy. But the biological importanc e of mitophagy in tumor progression and treatment response has been neglected for a long time, although with emerging clinical significance (particularly to cancer therapy) of it in recent years, the relationship between mitophagy and cancer has received increasing attention. Of interest, dual effects of mitophagy during tumor progression are largely modulated by the stage of tumor, histopathological subtype and mutation status, and the particularities of the TME 119.

### 3.1. Tumor-Suppressive Effect of Mitophagy in Cancer

The cancer-inhibitory function of mitophagy results from selective autophagic clearance of impaired mitochondria or parts thereof, and its regulation depends on a complicated regulatory network at multiple levels including signal transduction, post-translational modification and transcriptional control. In the well-known PINK1-Parkin cue, tumor suppressor activity of Parkin is connected to its key role in regulation of mitophagy and mitochondrial homeostasis. ROS are largely produced from damaged mitochondria, and abnormal mitophagy leads to an increase of ROS that may trigger subsequent signaling cascades for tumor cell survival 120,121. In multiple types of tumors, when essential mitophagy regulators are either deleted or inactivated, normal mitophagic activity is disrupted and damaged mitochondria accumulate. The tumor suppressor gene PARK2, situated in the q25.2-q26 on chromosome 6 is a classical tumor suppressor in solid tumors (including liver cancer and colorectal cancer) that loss of PARK2 function disrupts the mitochondrial ubiquitin-protein ligase system autophagy pathway, resulting in the accumulation of excessive ROS activation of NF-κB inflammatory axis, which significantly promoting tumor cell proliferation, invasion and metastasis 122,123. These results indicate that mitophagic action is critical for the suppression of tumorigenesis.

Beyond maintaining ROS homeostasis, mitophagy is able to coordinately adjust the cellular energy platform via metabolic flux reprogramming, compromising tumorigenesis. Mitophagy, via the PINK1-Parkin pathway, not only removes malfunctioning mitochondria but also terminates an abnormal increase in organelles by down-regulating expression of the fusion protein MFN2. At the same time, mitophagy inhibits hypoxia-inducible factor-1α (HIF-1α)/BNIP3 axis to resist Warburg effect, and cancer cells experience metabolic reprogramming from glycolysis into aerobic oxidation phosphorylation in which ATP production is less efficient than that in glycolysis 124. The metabolic reprogramming is orchestrated by specific regulation on two crucial enzymes: PINK1 ubiquitinates pyruvate dehydrogenase back to initiate TCA cycle flux and Parkin phosphorylates p53 to suppress stemness factors such as OCT4 and SOX2, resulting in TME dysfunction and impaired self-renewal of the tumor 125-127. Moreover, a positive feedback loop is established between mitophagy and metabolic remodeling. In this loop, metabolites released from cleared mitochondria activate HIF-1α and upregulate antioxidant gene expression to stabilize metabolic homeostasis 128. Clinically, in HCC, overexpression of the mitophagy receptor low molecular mass polypeptide 7 (LMP7) enhances fatty acid β-oxidation, elevates ROS production, and activates PINK1-Parkin signaling to drive lipid droplet biogenesis, developing chemoresistance. Targeted inhibitors (e.g., FL3) that selectively block LMP7-driven lipogenesis can reverse the metabolic reprogramming and restore normal energy metabolism, which could be a promising strategy to overcome therapeutic resistance 129, 130.

### 3.2. Oncogenic Effect of Mitophagy in Cancer

Tumor-derived oncogenic mitophagy is a defensive adaptive strategy. Under microenvironmental stress, such as hypoxia or nutrient deprivation, tumor cells maintain metabolic homeostasis and evade apoptosis by moderately activating the PINK1-Parkin and FUNDC1 pathways. In stark contrast, therapeutically induced excessive mitophagy utilizes exogenous means, such as drugs or nanomodulators, to exceed the physiological boundaries of autophagic flux. This powerful intervention triggers massive mitochondrial degradation and a complete collapse of cellular bioenergetics, fundamentally bypassing the regulatory network of tumors by directly damaging mitochondria or excessively activating the autophagy pathway. Mitophagy promotes malignancy in several human cancers via different aspects, such as metabolic reprogramming, immune escape and therapy resistance. Clinical research results of some diseases, which include glioblastoma, liver cancer, breast cancer and colorectal carcinoma coincide with this pro-tumor mechanism; for example, the primary human glioblastoma cells by PINK1-Parkin axis keeps the integrity of mitochondrial network and establishes stem-cell like phenotypes while FUNDC1 expression is 5.8-fold more in cisplatin-resistant ovarian tumor cells which can lead these cells to escape from immune surveillance 131.

Mitophagy may provide dynamic metabolic reprogramming for TME adaptation to stress. In colorectal cancer, mitophagy plays a role in the tumor-cell growth and survival by modulating glutamine metabolism. Under the shortage of nutrients, mitophagy is exerted and PTINKl/Parkin-mediated pathway was activated to remove damaged mitochondria; during this process, the expression of the glutamine transporter alanine/serine/cysteine ​​transporter 2 (ASCT2) is upregulated, enhancing the ability of tumor cells to take up glutamine. Intracellular glutamine is processed through glutamine catabolism in a linked series of enzymatic reactions to supply ATP and precursors for the biosynthesis of cells that promote tumor cell proliferation and survival​; meanwhile, mitophagy can suppress the activities of enzymes involved in glutamine metabolism, such as those described above, by rebuilding their substrates to increase the efficiency of glutamine degradation needed to meet metabolic demands within tumors despite nutritional stress and inducing tumorigenesis. In breast cancer cells, mitophagy allows tumor cells to metabolically adapt, by eliminating damaged mitochondria and shifting resources toward more efficient metabolic programs that promote their survival as well as metastatic potential. For instance, the removal of dysfunctional mitochondria in tumoural cells diminishes ROS generated by impaired ETCs thereby shielding cells from oxidative stress-induced injury and it providing them with energy-expensive tasks like cellular motility and proliferation 132. The finding is consistent with observations that mitophagy can remove chemotherapy-induced mitochondria damage and prevent cytochrome c to be released, meanwhile, results in increasing the expression of solute carrier family 38 member 2 (SLC38A2), and contributing to the survival under nutrient deprivation by promoting glutamine-dependent synthesis for biosynthesis in pancreatic adenocarcinoma 133-135.

Mitophagy fortifies tumor resistance by clearing therapy-induced mitochondrial damage.​​ In cisplatin-treated cells, cisplatin-induced mitochondrial membrane depolarization triggers ​ATG5/Beclin-1-dependent​ mitophagy. This process reduces the transfer of the pro-apoptotic protein BAX from the cytoplasm to the mitochondrial membrane and ​prevents​ mitochondrial membrane permeability transition and subsequent cytochrome c release, ultimately enhancing​ tumor cell survival 136, 137. ​Notably, in cisplatin-resistant cell lines, the FUNDC1 expression level increases 5.8-fold compared to sensitive counterparts. Under hypoxic conditions, FUNDC1 interacts with the ATG LC3 to mediate mitophagy. ​Through​ clearing damaged mitochondria and maintaining mitochondrial homeostasis, ​the FUNDC1-mediated mitophagy​ may contribute to enhanced tolerance of tumor cells to cisplatin. Inhibition of the FUNDC1-mediated mitophagy axis significantly reduces the half-maximal inhibitory concentration (IC_50_) of cisplatin 138, 139. In another study, in taxane-resistant breast cancer cells, mitophagy preserves MFN2-mediated mitochondrial fusion and suppresses Drp1-driven fission, which reduce the production of damaged mitochondria, decrease apoptosis risks, and promote tumor cell survival. ​Notably, Mdivi-1, a small-molecule inhibitor that pharmacologically targets Drp1, can reverse drug resistance. It has been demonstrated that Mdivi-1 treatment restores paclitaxel sensitivity and increases apoptosis rates in taxane-resistant breast cancer cells 140, 141. In head and neck squamous cell carcinoma, radiotherapy-triggered mitophagy eliminates excess ROS and inhibits caspase-9 activation. ​Patients with a high LC3B-II expression level (a key autophagy/mitophagy marker) after radiotherapy exhibit a three-year recurrence rate of 61%, compared to 29% in those with a low LC3B-II expression level 142, 143. Collectively, these findings underscore the pivotal role of mitophagy in overcoming therapeutic resistance and ​support its dynamic regulation could be a promising strategy for ​combination therapies.

Mitophagy also reshapes an immunosuppressive TME through metabolite-mediated regulation of immune cells. In glioblastoma, PINK1-Parkin-driven mitophagy releases succinate, which engages the triggering receptor expressed on myeloid cells 2 (TREM2), polarizes macrophages to an M2 phenotype, induces IL-10 and TGF-β secretion, and impairs CD8^+^ T cell infiltration 144, 145. In non-small cell lung cancer, mitophagic clearance of mtDNA attenuates cGAS-STING signaling and type I interferon production, facilitating NK cell evasion. Silencing PINK1 in A549 cells reduces cancer-associated fibroblast (CAF)-derived IL-6 secretion by 40% and reverses local immunosuppression 146-148. Moreover, in HCC, mitophagy-driven, Parkin-mediated NF-κB activation upregulates PD-L1. Patients with high PD-L1 expression exhibit an objective response rate of only 12% to immune checkpoint inhibitors, compared to 34% in the PD-L1 low-expression cohort, suggesting there is a strong link between mitophagy and immune resistance 149, 150.

**Figure 4:**
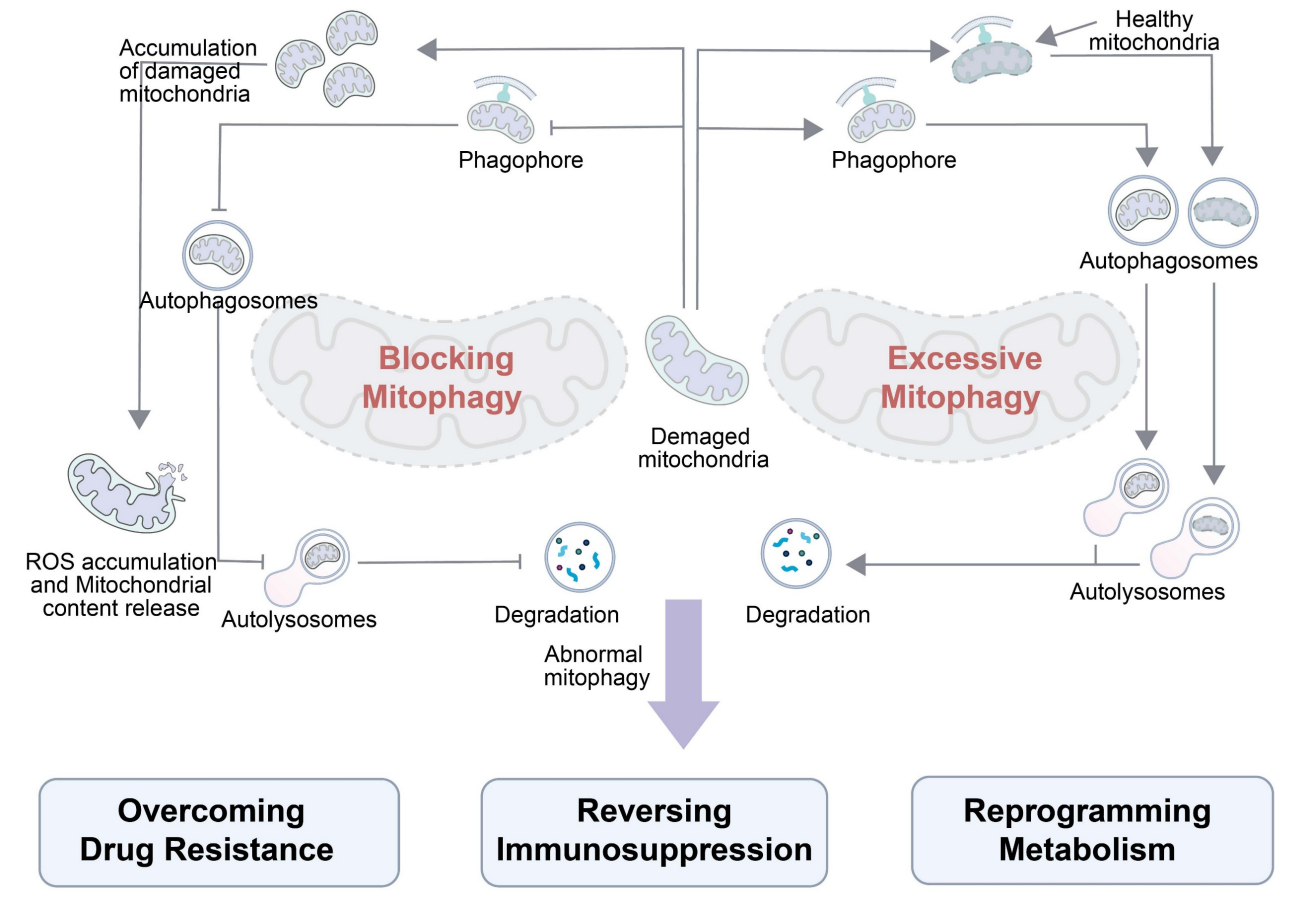
Mitophagy holds significant therapeutic potential in oncology by leveraging two complementary strategies: blocking mitophagy to reverse immunosuppression and overcome drug resistance, and excessively inducing mitophagy to reprogram metabolism. Created in https://BioRender.com.

Hence, mitophagy holds significant therapeutic potential in oncology by leveraging two complementary strategies (**Figure 4**): blocking mitophagy to reverse immunosuppression and induce ROS-mediated damage, and inducing excessive mitophagy to reprogram tumor metabolism.​​ When mitophagy is blocked within tumor cells, damaged mitochondria accumulate, rather than degrade, ​triggering​ excessive ROS production and mitochondrial content release and ​undermining the tumor immunosuppressive microenvironment. The reshaped microenvironment enhances the efficacy of anticancer therapies and aids in overcoming drug resistance. By contrast, inducing excessive mitophagy results in degradation of both healthy and damaged mitochondria. Phagophores engulf these mitochondria to form autophagosomes, which are fused with lysosomes for degradation. ​Excessive degradation​ of mitochondria disrupts the metabolic balance in tumor cells, ​leading to metabolic reprogramming​ of their energy pathways. Together, these dual mitophagic approaches can exploit tumor vulnerabilities, suggesting manipulating MQC​​ can harness the tumor survival mechanisms for therapeutic gain. Ultimately, there is a profound fundamental difference between blocking mitophagy and inducing excessive mitophagy. The former constitutes a “passive defense mechanism” for tumor survival, while the latter is a “therapeutic strategy” that actively disrupts the metabolic balance of the tumor. The differences in the activation intensity and regulatory pathways between blocking mitophagy and inducing excessive mitophagy lead to distinct biological effects.

### 3.3. Small-Molecule Regulation of Mitophagy in Cancer

**Figure 5:**
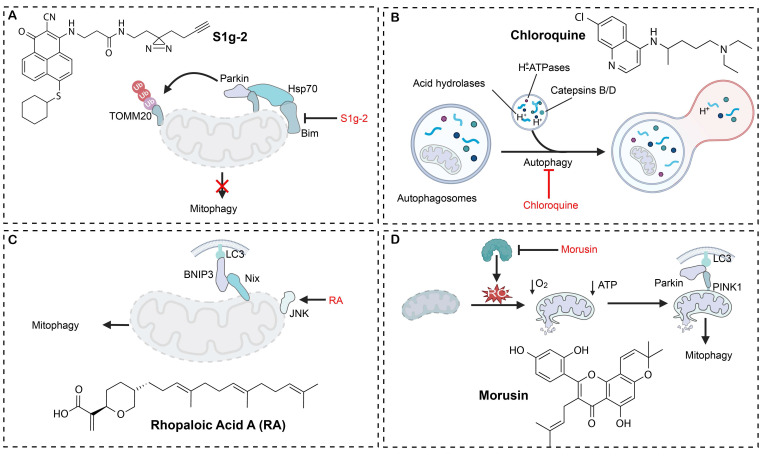
Mitophagy regulatory mechanisms for four typical compounds. (A) S1g-2 inhibits mitophagy by acting on proteins including Parkin and Hsp70. (B) Chloroquine blocks autophagic degradation by interfering with the fusion of autophagosomes and lysosomes. (C) RA activates the JNK pathway and promotes mitophagy via proteins such as BNIP3 and Nix. (D) Morusin induces mitophagy by elevating ROS and ATP levels to induce mitochondrial damage and activating the PINK1-Parkin pathway. Created in https://BioRender.com.

Mitophagy modulators​ have emerged as a novel anticancer drug candidate, and remarkable strides have been made in mechanism-based targeting and clinical translation of mitophagy modulators. Small-molecule regulators have been developed to selectively engage key checkpoints of mitophagy and they have exhibited precise regulatory potential across diverse tumor models (**Table 1**). A notable example is S1g-2, the first targeting inhibitor of the Hsp70-Bim complex, which blocks mitophagy-mediated apoptotic escape and induces over 50-fold tumor suppression greater than imatinib in chronic myeloid leukemia (**Figure 5A**) 151. ONX0912, another modulator targeting the Parkin-PINK1 axis, potentiates mitophagy and induces apoptosis, markedly reducing the viability of HCC cells 25. Based on their mechanistic profiles, mitophagy modulators are often classified into two functional categories: mitophagy inducers and mitophagy inhibitors.

A few natural compounds have exhibited distinctive antineoplastic activities by modulating mitophagy pathways. Costunolide and tanshinone I can elevate the intracellular ROS level by activating the ROS-AKT and ROS-ERK signaling axes to trigger HCC cell death. Concurrently, these agents downregulate PINK1 expression and suppress PINK1/Parkin-mediated mitophagy, thus promoting apoptosis and inhibiting tumor cell proliferation 26. Moreover, soy isoflavones (SI) could trigger mitophagy in a dose-dependent manner as significant increase of mitochondrial autophagosomes can be found and accompanied by the collapse of MMP (loss), the upregulation of autophagy-related markers and the change in oxygen consumption at lung cancer cells. This increased mitophagy was correlated with a dramatically decrease of tumor cell viability, clonogenic ability, migration and invasion capability and an increase of apoptosis. Soy isoflavones achieve these effects at the molecular level by blocking AKT/mTOR signaling. This observation indicates the therapeutic implications of bioactive compounds obtained from plant-origin towards the regulation of MQC and cancer cell growth inhibition 152.

Dynamically inducing mitophagy is important for sensitizing the effects of chemotherapeutics. Statins (atorvastatin) may trigger mitochondrial injury and apoptosis in tumor cells, yet tumors frequently become resistant to these agents by increasing autophagic removal of damaged mitochondria. Clinical cohort analysis reveals mitophagy as a key mechanism of statin resistance. In patient-derived organoids, organoid derived xenograft models and AOM/DSS-induced CRC mice model, both genetic and pharmacological inhibition of mitophagy dramatically increases the anti-tumor effects of statins 153. These findings further validate the resistance to AICAR induced cell death, confirm that the inhibition of mitophagy would be a novel strategy to increase the individual difference in statins response of colorectal cancer patient and outcome. The novel oral proteasome inhibitor ONX0912 is proved to induce mitophagy activation as its central antitumor mechanism for liver cancer therapy: It activates the PINK1/Parkin/p62 pathway, resulting in mitochondrial membrane depolarization and reactive oxygen species (ROS)-mediated apoptosis; moreover, silencing of the related mitophagy receptor p62 significantly intensified this apoptotic effect, indicating the precise interplay between mitochondria-selective autophagy and cell death signaling. These data demonstrate the dual action of ONX0912 for autophagy regulation and induction of apoptosis, which indicates that mitophagy may be a feasible strategy in liver cancer therapy 25.

Chloroquine derivatives reverse drug resistance by blocking the acidification of lysosomes, hence they are frequently employed to potentiate the effect of chemotherapeutics in breast cancer. In a PI3K/AKT-activated TNBC resistance model, ipatasertib and taselisib paradoxically intensify the autophagic flux, leading to treatment failure. Conversely, chloroquine-mediated autophagy blockade effectively reverses the resistance of ipatasertib and taselisib, markedly boosting the antitumor activity of PI3K/AKT inhibitors in TNBC cell lines and *in vivo* models. Chloroquine derivatives in synergy with taxane chemotherapy have significantly inhibited tumor progression and overcome chemotherapeutic drug resistance in the TNBC resistance model (**Figure 5B**). These results confirm that mitophagy inhibition could be a viable strategy to surmount PI3K/AKT inhibitor-associated resistance in TNBC, and compelling preclinical data supports clinical translation of chloroquine derivatives 154. DC-ATG4in, a small-molecule inhibitor, has been discovered to directly bind to ATG4B protease and inactivate its activity via a novel high-throughput screening platform. By obstructing autophagosome maturation, DC-ATG4in significantly augments the antitumor efficacy of sorafenib in HCC models. Co-administration of DC-ATG4in with sorafenib synergistically suppresses tumor cell proliferation and effectively overcomes acquired resistance, therefore, targeting ATG4B could be a promising strategy to sensitize tumors for established targeted therapies 155.

Targeting metabolic reprogramming offers an additional critical strategy for therapeutic intervention. By activating the miR-26a-5p/DAPK1 axis, rapamycin drives enhanced clearance of damaged mitochondria, resulting in profound suppression of tumor growth in glioma models 156. Retinoic acid (RA) specifically inhibits the survival of oral squamous cell carcinoma (OSCC) cells without affecting normal epithelium, induces chromatin condensation and increases expression of selective mitophagy and apoptosis regulators. Simultaneously, RA also activates the JNK/BNIP3/Nix/LC3B axis and eventually leads to marked mitochondrial damage resulted with excessive mitophagy followed by tumor cell death (**Figure 5C**). Zebrafish xenograft assays confirm that RA treatment markedly inhibits OSCC progression, revealing a synergistic interplay between mitochondrial injury-driven apoptosis and mitophagy, which could be a novel therapeutic mechanism for treating OSCC 157. Morusin (Mor) directly binds to the catalytic domain of the ATP citrate lyase (ACLY), resulting in a marked reduction in the ACLY expression and its enzymatic activity. The disruption of the metabolic pathway leads to intracellular ROS accumulation and mitochondrial dysfunction, which in turn activates PINK1/Parkin-mediated mitophagy and triggers intrinsic apoptotic pathways in HCC cells (**Figure 5D**). It has been shown that the proapoptotic action of Mor could be abrogated by ROS scavengers. Analysis of clinical specimens reveals a positive correlation between the ACLY level and the HCC histopathological grade, suggesting that Mor can induce metabolic reprogramming-mediated apoptosis, and it holds significant promise as an antineoplastic agent 158.

In addition to the above-mentioned mitophagy-targeting small molecules, a myriad of low-molecular-weight compounds has shown potent modulatory effects on mitophagy across diverse tumor models, therefore, they could be harnessed to enhance the efficacy of antineoplastic therapies, and a few of them are listed in **Table 1**. Collectively, these modulatory strategies exploit distinct molecular targets and enable dynamic regulation of the mitophagic flux to provide innovative approaches for overcoming therapeutic resistance.

### 3.4. Targeting Mitophagy Regulation Using Nanotherapeutics

Despite the promising anticancer potential of small-molecule mitophagy agents, their clinical translation remains hampered due to their poor target specificity and severe systemic toxicity. Conventional agents disrupt core mitophagy axes (e.g., the PINK1-Parkin pathway) through non-selective mechanisms, which may lead to aberrant clearance of healthy mitochondria and severe adverse effects 167. Additionally, most agents exhibit suboptimal physicochemical properties. Natural products and their synthetic derivatives suffer from poor aqueous solubility and low metabolic stability, resulting in inadequate bioavailability. Meanwhile, synthetic peptides or hydrophilic small molecule inhibitors suffer from low membrane permeability, making it challenging to reach therapeutic drug levels in tumors. To overcome this challenge, multi-modal approaches are under constant refinement, which includes bifunctional constructs like AUTACs that selectively eliminate dysfunctional mitochondria by engaging ubiquitin-proteasome system without causing a global activation of the same 29. In addition, more and more studies focus on the design of mitochondria-targeting delivery systems to promote the specificity of MPTP regulators toward tumor mitochondria for targeted therapy while also reducing systemic exposure and extratumor toxicity, thus improving therapeutic efficacy 168. These groundbreaking advances provide an important basis for future clinical application of anticancer drugs that target mitophagy.

#### 3.4.1. Liposomes for mitophagy regulation

Liposomes represent one of the dominant classes of nanocarriers in translational oncology by virtue of their high biocompatibility and negligible intrinsic toxicity. All is centered in the maintenance of therapeutically active drug concentrations at needed site and in achieving specific subcellular delivery (especially to mitochondria) involved in governing signal cascades, calcium homeostasis, and energy metabolism. Although mitochondria-targeted drug delivery systems are desired, their clinical applications have been hindered.

Mitochondria-targeted liposomes represent an innovative option compared with conventional delivery systems and can reduce non-specific interactions and toxicities due to the rational design of carriers as well as target ligands. This liposomal approach unifies multidisciplinary technologies in chemical engineering (ligand conjugation), pharmacology (mitophagy control and pharmaceutics formulation, optimization), and cell biology (mitochondrial trafficking and function). It effectively improves efficacy and safety at a lower dose or with less frequent administration by improving specificity in targeting. Moreover, the co-encapsulation of mitophagy regulators with clinically-used anticancer drugs into this liposomal formulation serves to show a synergistic tumor inhibition, presenting an extremely attractive approach for controlling intra-tumor diversity and drug resistance.

**Figure 6:**
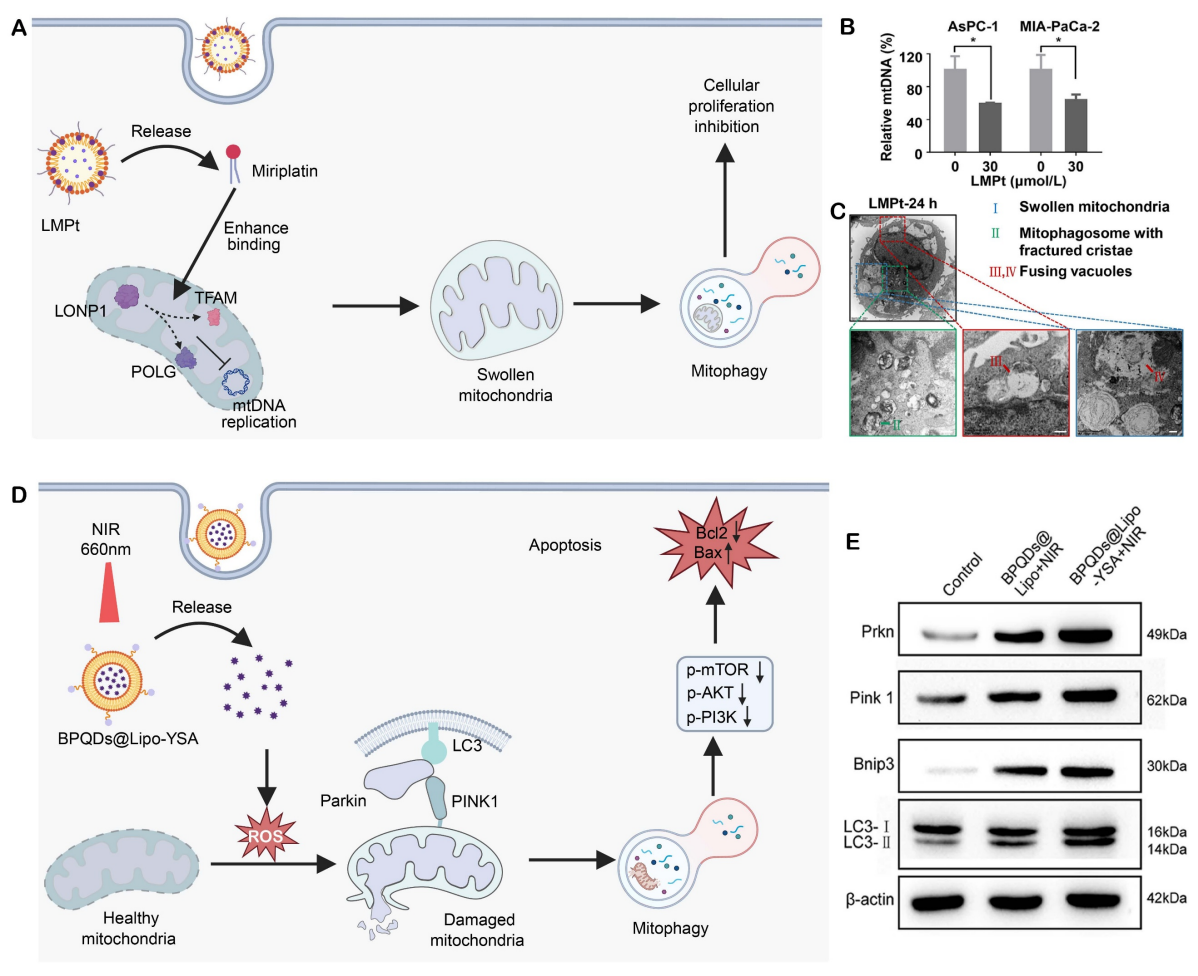
(A) Miriplatin, released from LMPt, enhances binding of factors like LONP1 to TFAM and POLG, and interferes with mtDNA replication via TFAM and POLG, leading to swollen mitochondria and subsequent mitophagy and inhibition of cellular proliferation. Created in https://BioRender.com. (B) Relative mtDNA levels in AsPC-1 and MIA-PaCa-2 cells after LMPt treatment. (C) Transmission electron microscopy images of mitochondria at 24 h post-LMPt treatment. Swollen mitochondria, mitophagosomes with fractured cristae, and fusing vacuoles are observed. Adapted with permission from 169, copyright © 2023, Elsevier. (D) BPQDs@Lipo-YSA, released under NIR 660 nm irradiation, induces ROS generation to damage mitochondria, activates Parkin-PINK1-mediated mitophagy, and ultimately promotes apoptosis via downregulating p-mTOR, p-AKT, and p-PI3K and upregulating Bax/Bcl2. Created in https://BioRender.com. (E) Western blot analysis of apoptosis-related proteins (Bax, Bcl-2, p53 and Cle-caspase-3) in different treatment groups. Adapted with permission from 170, copyright © 2025, Springer Nature.

Pancreatic cancer, which is one of the most aggressive malignancies, has extremely limited therapeutic options because of drug resistance and systemic toxicity, recent advances have been rare in the field of treatment for pancreatic cancer. One of these methods was introduced by Wang, C, et al. They designed a mitophagy regulatory miriplatin-loaded liposomes (LMPt) that could target the mitochondria (**Figure 6A**) 169. ​Due to​ the structural similarities of long myristoyl chains in miriplatin and phospholipids, LMPt has a good drug loading capacity (19.04%) and robust structure stability. LMPt facilitate cellular uptake and multi-pathway endocytosis is found to be mediated largely by caveolin-1 (Cav-1). 53.8%-62.9% of the intact MPt specifically accumulates in mitochondria following lysosomal release by the endosomal-lysomal system. This result radically changes the previous concept that platinum-based drugs predominantly target the cell nucleus. When localized into mitochondria, MPt did not cause mtDNA damage but it promoted LON protease 1 (LONP1) binding to the two master regulators of mtDNA replication: POLG and TFAM. By increasing the interaction, MPt facilitates degradation of POLG and TFAM, inhibiting mtDNA replication (**Figure 6B**).

In case of mtDNA repair inhibition, MPt recruits PINK1 to the mitochondria and ubiquitinates it along with phosphorylating Parkin, resulting in activation of the PINK1-Parkin pathway with subsequent induction of mitophagy (**Figure 6C**). Importantly, the autophagy inhibitors 3-methyladenine (3-MA) and bafilomycin A1 (Baf-A1) dramatically rescued the anti-tumor effect of LMPt. *In vivo* studies demonstrated the LMPt did not have the side effects including peripheral neurotoxicity and myelosuppression of traditional platinum compounds. Meanwhile, such treatment worked efficiently with low toxicity. This study elucidates the core role of the LONP1-POLG/TFAM-mtDNA-PINK1-Parkin axis in liposome-mediated mitophagy regulation, suggesting that POLG and TFAM may promote pancreatic cancer progression by inhibiting mitophagy.

Lung adenocarcinoma, a predominant subtype of non-small cell lung cancer (NSCLC), is characterized by a high incidence, significant therapeutic resistance, and poor patient outcomes. Emerging evidence supports that modulating mitophagy can reprogram tumor cell susceptibility to therapeutic treatment. Recently, a mitochondria-targeting photosensitizer nanocomposite, BPQDs@Lipo-YSA, was constructed and characterized by Li et al. This uniform-sized nanocomposite​ induces mitochondrial membrane depolarization and activates Parkin/AKT1-mediated selective mitophagy upon near-infrared laser irradiation (**Figure 6D**) 170. The enhanced mitophagic flux triggers pro-apoptotic signaling, including upregulation of Bax and downregulation of Bcl-2 (**Figure 6E**), and promotes the release of damage-associated molecular patterns (DAMPs, molecules released by dying cells that trigger immune responses), which enhance dendritic cell maturation and T-cell activation. In tumor-bearing mice, BPQDs@Lipo-YSA induces immunogenic cell death, enhances intratumoral CD8^+^ T-cell infiltration and inhibits tumor growth. ​These findings indicate that combination of mitophagy regulation with immune activation could be a novel approach for lung cancer therapy.

Liposomal nanocarriers modified with targeting ligands can precisely anchor to tumor mitochondria to enhance the delivery efficiency and specificity of mitophagy-regulating molecules and reduce their side effects on normal cells. A mitochondria-dual-targeting nanosystem, TPP-SS-ATS-LS, was development by surface-conjugating with triphenylphosphonium (TPP) and glucose moieties via a glutathione-sensitive disulfide linker. Artemisinin (ATS) is selectively released from the nanosystem in the TME. After cellular uptake of the nanosystem, the released ATS downregulates Prohibitin 2 and activates PINK1-mediated mitophagy, thereby potently promoting breast cancer cell death 171. While liposome-induced mitophagy predominantly triggers tumor cell apoptosis, growing evidence support that it can also initiate alternative death modes like ferroptosis via mitochondrial homeostasis disruption. For example, a nanoparticle delivery system was developed by encapsulating a G protein-coupled estrogen receptor 1 (GPER1) agonist, G-1, in liposomes. G-1 activation of GPER1 triggers mitochondrial dysfunction and induces ferroptosis, markedly suppressing GL261 and U251 glioma cell proliferation. Mechanistic studies reveal this process involves upregulating lipid peroxidation pathways mediated by an imbalance between acyl-CoA synthetase long chain family member 3 and glutathione peroxidase 4 and activating autophagy markers (e.g., LC3-II, ATG7), ultimately leading to cell death through mitochondrial membrane depolarization and iron accumulation 172. In another study, a novel liposomal nanomedicine (CD-NDs) was fabricated via thin-film dispersion by co-encapsulating an antibiotic, doxycycline (Doxy), and a photosensitizer, chlorin e6 (Ce6). Mechanistically, CD-NDs reduces the mitochondrial membrane potential, disrupts the mitochondrial morphology, and amplifies ROS production, consequently potentiating photodynamic therapy (PDT)-mediated cell killing. Concurrently, CD-NDs upregulates p62 expression and inhibits LC3-II conversion, thereby blocking degradation of MHC-I (major histocompatibility complex class I). The enhanced MHC-I surface expression on tumor cells promotes antigen presentation and cytotoxic T lymphocyte (CTL) recognition. These findings confirm that targeting mitophagy enhances photoimmunotherapy efficacy 173.

Despite substantial antitumor benefits of these mitochondria-targeting liposomal nanosystems, their design is often relied on the mitochondrial membrane potential and these nanosystems have high membrane affinity. Such high affinity may result in perturbing the mitochondrial structure and impairing the respiratory chain or ATP synthesis. The targeting specificity and efficiency of these nanosystems may be compromised due to the mitochondrial membrane potential (ΔΨm) fluctuations within the pathological microenvironment. ​To address these limitations, future innovations should be shifted towards ΔΨm-independent targeting strategies, such as engineering exosome-membrane-modified liposomes to harness endogenous mitochondrial tropism or designing MFN1/2 fusion proteins to mimic native protein translocation via the TOM/TIM complexes.

#### 3.4.2. Polymers for mitophagy regulation

Polymers, a versatile and efficient drug delivery platform, offer another breakthrough in targeted delivery of mitophagy modulators, owing to their unique physicochemical properties and tailorable chemical/physical functionalities.​​ ​Notably, polymers, including poly(lactic-co-glycolic acid) (PLGA), amphiphilic polyethylene glycol (PEG) derivatives, and natural polymers (e.g., chitosan, hyaluronic acid, and heparin), enable precise mitochondrial targeting through ​surface functionalization strategies, such as covalent conjugation of mitochondria-targeting peptides or lipophilic cations. These modifications leverage a negative charge of mitochondrial membranes or an enzyme-specific cleavage site to enhance drug accumulation in the mitochondrial vicinity. ​Additionally, biocompatibility and biodegradability of polymers significantly reduce their systemic toxicity, while their controlled-release properties extend the duration of modulation action of mitophagy modulators and reduce their administration frequency. ​By harnessing recently revealed mitophagy mechanisms and advances in polymer materials science, efficient, safe, and precise therapeutic approaches based on polymer-based delivery systems have been developed, ​and they offer new avenues to improving outcomes for clinical cancer treatment.

**Figure 7:**
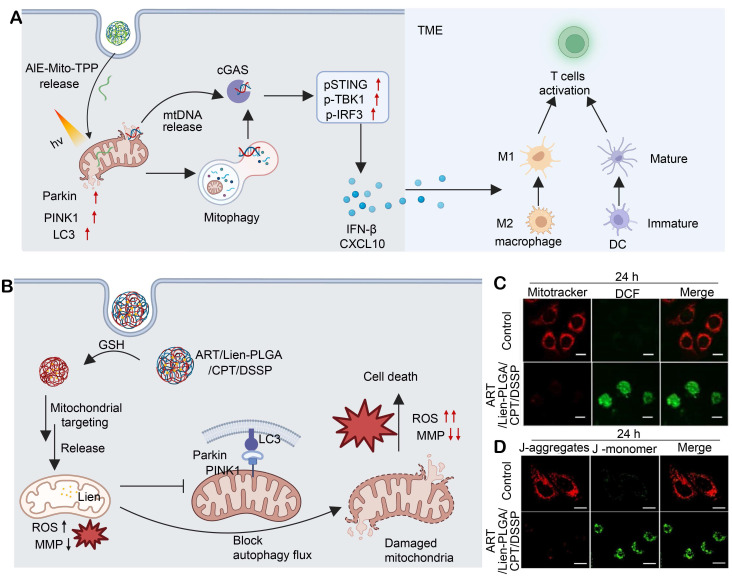
(A) AIE-Mito-TPP, upon light stimulation, induces mitophagy and promotes mtDNA release, which activates the cGAS-STING pathway to produce IFN-β and CXCL10, thereby activating dendritic cells (DCs) and M2 macrophages and promoting T cell activation. Created in https://BioRender.com. (B) Lien, released from ART/Lien-PLGA/CPT/DSSP after targeting mitochondria that is aided by glutathione (GSH), blocks the autophagy flux via Parkin/PINK1/LC3, leading to damaged mitochondria, increased ROS, decreased MMP, and ultimately cell death. Created in https://BioRender.com. (C) Fluorescence images of MitoTracker and DCF (for ROS detection) in different treatment groups at 24 h. (D) Fluorescence images of J-aggregates and J-monomers in different treatment groups. Adapted with permission from 182, copyright © 2023, American Chemical Society.

It has been reported that mtDNA released under mitochondrial stress activates innate immunity via the cGAS-STING pathway 174, and agonists including palmitic acid and cisplatin induce cytosolic mtDNA leakage to trigger STING signaling 175, 176. However, cellular repair programs, including mitochondrial fission/fusion dynamics and mitophagy, clear damaged mtDNA and weaken the activation 177. To address this issue, a polymer nanoplatform, DPPA-1 M@AIE-Mito-TPP, was engineered by encapsulating AIE-Mito-TPP within a PLGA-PEG-maleimide backbone functionalized with DPPA-1 M, a metalloproteinase (MMP)-2/9-responsive PD-L1 peptide. ​This nanoplatform displays prolonged systemic circulation, enhances its accumulation in HCC tissues, and enables mitochondrial targeting 178. AIE-Mito-TPP triggers autophagy but blocks mitochondrial fusion, leading to incomplete mitophagy and mtDNA release to activate cGAS-STING, which is evidenced by TBK1/STING/IRF3 phosphorylation and IFN-β secretion. Activation of cGAS-STING also promotes antigen release in Hepa1-6 cells, induces DC maturation, elevates macrophage NF-κB, and drives M2-to-M1 macrophage polarization, collectively supporting CD8⁺ T cell-mediated antitumor immunity (**Figure 7A**).

Artemisinin (ART) has displayed the anticancer potential across multiple tumor types 179. However, its therapeutic efficacy remains disappointing. Mitochondria-targeting delivery systems for ART have enhanced ART-induced mitochondrial damage in cancer cells, while resistance develops at an elevated concentration of ART, primarily attributed to mitophagy. In this process, damaged mitochondria are selectively cleared via the PINK1-Parkin pathway and LC3-II-dependent autophagosome formation, attenuating cytotoxic effects of ART 180. Liensinine (Lien), an autophagy inhibitor, has been found to interrupt the mitochondrial repair pathway and synergistically enhance ART-induced apoptosis 181. Li et al. developed a mitochondria-targeting polymeric nanoplatform, ART/Lien-PLGA/CPT/DSSP, by co-assembling ART and Lien with PLGA, C18-PEG2000-TPP (CPT) and DLPE-S-S-mPEG4000 (DSSP) (**Figure 7C**) 182. The self-assembled system forms uniform-sized, spherical nanoparticles at approximately 150 nm in diameter and exhibits glutathione-responsive release kinetics. Under a mildly acidic lysosomal environment, surface-charge reversal unmasks TPP cations to enhance mitochondrial uptake of ART/Lien-PLGA/CPT/DSSP. Within the mitochondrial matrix, heme-mediated activation of ART generates ROS, triggering mitochondrial membrane depolarization, electron transport chain dysfunction, and organelle fragmentation (**Figure 7D and 7E**). Simultaneously, Lien inhibits autophagosome-lysosome fusion, blocks PINK1-Parkin-mediated mitophagic clearance and amplifies oxidative injury. In an MCF-7 xenograft model, treatment with ART/Lien-PLGA/CPT/DSSP nanoparticles achieves superior tumor growth inhibition (88.8% vs. 75.8% with ART alone). This polymer-based dual-drug delivery system overcomes the resistance of ART by blocking mitophagy and achieves dose reduction, providing a safe and effective strategy for the treatment of breast cancer.

Since lung carcinoma has high mortality and conventional chemotherapy is often accompanied with systemic toxicity, an inhalable PLGA-PSPE polymeric nanocarrier was developed for pulmonary delivery of dihydroergotamine (DHE). After synthesis via Michael addition, the copolymer self-assembles into DHE-encapsulating nanoparticles that target respiratory epithelium upon aerosolization. Upon internalization by tumor cells, the nanoparticles induce apoptosis and activate DRP1/PINK1/Parkin-mediated mitophagy, potently inhibiting tumor growth in K-rasLA1 mice without systemic toxicity 183. Acute myeloid leukemia (AML) relapse is driven by drug-resistant leukemic stem cells (LSCs) in the bone-marrow niche. Cheng et al. engineered a bone-marrow-responsive polymer system, PPLFazo/siFis1@C. There are two payloads: a CXCR4 antagonist, plerixafor, to mobilize LSCs from the niche, and Fis1 siRNA to inhibit mitophagy-driven stemness maintenance. The polymer system in combination with chemotherapy synergistically eradicates LSCs, reprograms mitophagy signaling, extends 3.2-fold murine survival compared to chemotherapy alone, and reduces relapse 184.

Together, these studies highlight polymer-based mitochondria-targeting systems for co-loading therapeutics and mitophagy modulators. By integrating targeting and stimuli-responsive release, they achieve enhanced accumulation, efficacy, and minimal toxicity, offering promising strategies​ for treating treatment-resistant cancers.

#### 3.4.3. Other nano-delivery systems for mitophagy regulation

Hypoxic tumors can be adapted to an environment with chronic oxygen deprivation by reprogramming metabolic networks, such as augmenting glycolysis, stabilizing HIF-1α signaling, and upregulating survival pathways 181. Notably, hypoxia in the TME not only reduces the cellular viability but also disrupts mitochondrial homeostasis by activating PINK1/Parkin-mediated mitophagy. While the mitophagic response preserves energy homeostasis, mitophagy paradoxically drives therapeutic drug resistance in malignant cells 185-187. Consequently, selective modulation of mitophagy in hypoxic tumors has emerged as a promising therapeutic strategy to impair tumor survival and reverse drug tolerance.

**Figure 8:**
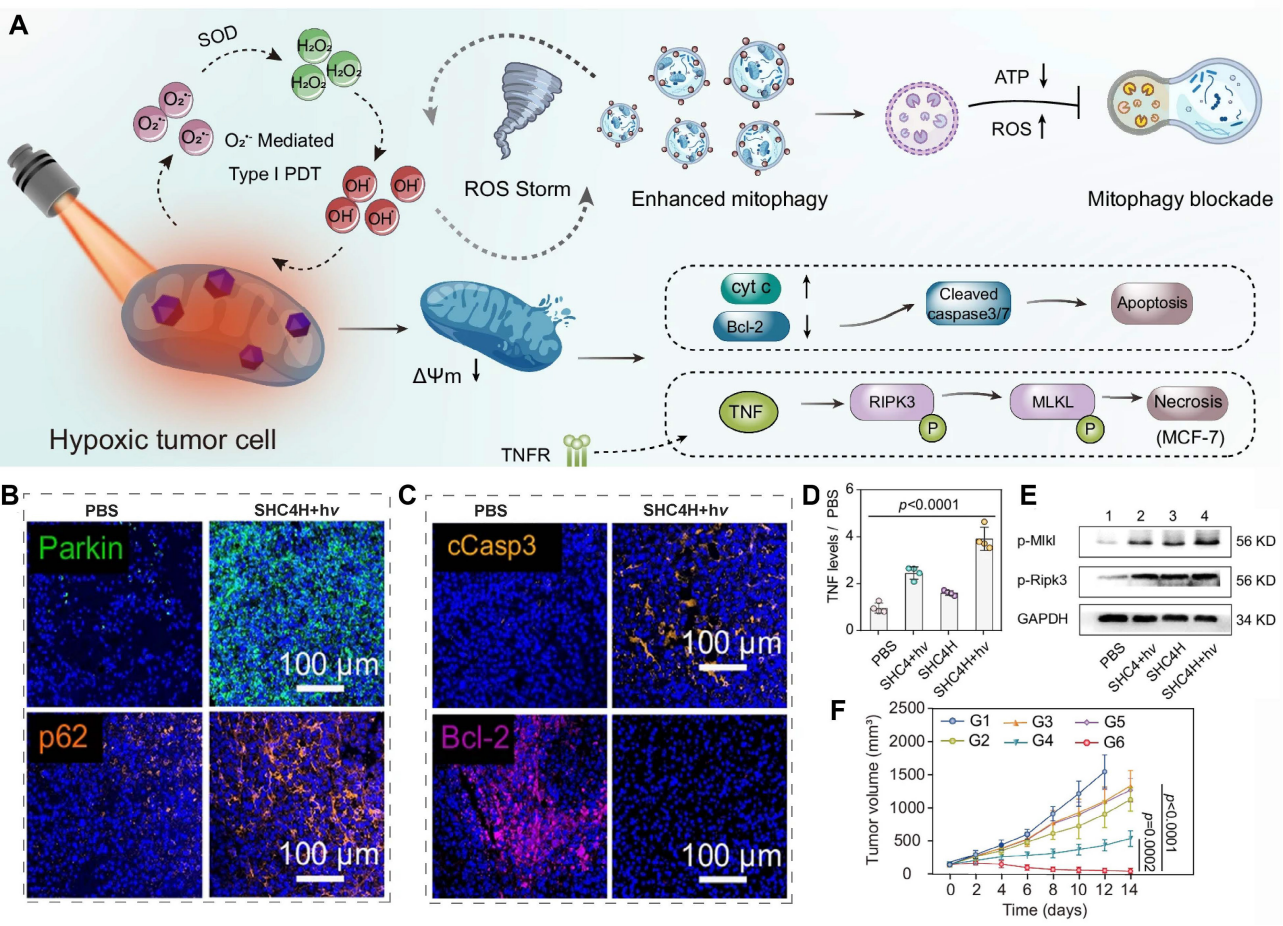
(A) Under light irradiation, type I PDT results in the generation of ROS like •OH. A ROS storm amplifies the loss of the ΔΨm, triggering mitophagy; mitophagy blockade by HCQ elevates the level of ROS and reduces the generation of ATP, thereby activating apoptosis (via cytochrome c, Bcl-2, cleaved caspase-3) and necrosis (via TNF, RIPK3, MLKL) pathways. Immunofluorescence staining of Parkin and p62 (B), cleaved caspase-3 and Bcl-2(C) in different groups. (D) TNF receptor levels in various treatment groups. (E) Western blot analysis of p-Mlkl and p-Ripk3. (F) Tumor volume changes over time in different treatment groups (G1. PBS, G2. HCQ+SMNB+hv, G3. HC4H, G4. SHC4 +hv, G5. SHC4H, G6. SHC4H+hv), and the group 6 displays the highest therapeutic efficacy. Adapted with permission from 188, copyright © 2025 Springer Nature.

Azobenzene-carbonyl alkyne-functionalized supramolecular albumin nanoparticles (SHC4H) were engineered to co-encapsulate hydroxychloroquine (HCQ) and SMNB, a mitochondria-targeting photosensitizer. The nanoparticles display a hypoxia-responsive drug release mechanism​ (**Figure 8A**). Upon cellular uptake, ROS is elevated after SMNB induction under light irradiation (SMNB+hν). Hypoxia and elevated ROS damage mitochondria and trigger mitophagy via upregulation of Parkin, while HCQ impairs the lysosomal function by alkalinizing lysosomes and inhibiting their enzymatic activity, which leads to the accumulation of P62 (rather than downregulation), thereby effectively blocking the degradation of damaged mitochondria and inducing mitophagic stress (**Figure 8B**). The stress results in the downregulation of Bcl-2, the generation of cleaved cCasp3 (**Figure 8C**), and the induction of apoptosis. Additionally, the stress can stimulate the secretion of TNF, induce the phosphorylation of MLKL and Ripk3 (**Figure 8D and 8E**), and trigger cell necrosis. Eventually, tumor growth is significantly inhibited (**Figure 8F**). As a result, this study not only introduces an innovative hypoxia-responsive drug-delivery platform but also establishes a promising therapeutic strategy for treating hypoxic malignancies by integrating mitophagy inhibition with photodynamic therapy 188.

In addition, alternative nano-delivery platforms have been explored for mitophagy modulation in cancer therapy, and many of these platforms leverage a hybrid system to integrate multiple functionalities. Sun et al. developed a lanthanide-protein-resveratrol hybrid (NCPRB). This hybrid can transfer near-infrared (NIR) energy at 808 nm to Ce6 and resveratrol is sequentially released from the hybrid, inducing a closed-loop photodynamic-immunotherapy response characterized by ROS-driven mitophagy, ferroptosis, and immunogenic cell death (ICD, a type of cell death that elicits adaptive immune responses) 189. Li et al. embedded Trop2-targeting chimeric antigen receptor T cells (CAR-T) and a mitophagy agonist, BC1618, into a gelatin methacrylate (GelMA) hydrogel. The hydrogel composite restores the mitochondrial function for ATP production and promotes cytokine secretion to reverse CAR-T exhaustion in TNBC xenografts 190. Lu et al. developed an LPT/HA-CD nanoprodrug. Upon GSH-triggered disassembly in CD44^+^ lung cancer cells, lonidamine and cisplatin (Pt(II)) are co-released to disrupt glycolysis/OXPHOS (oxidative phosphorylation), deplete GSH, and induce apoptotic and mitophagic death in cisplatin-resistant tumors 191. Zhou et al. designed PYT@ZIF8@siRNA by co-loading zinc ionophore pyrithione and SLC30A1 siRNA, a mitochondrial zinc transporter inhibitor, into zeolitic imidazolate framework-8 (ZIF-8). The platform overloads zinc ions in mitochondria and induces pro-death mitophagy and immunogenic cell death. Meanwhile, the platform eradicates intratumoral *Porphyromonas gingivalis*, a bacteria associated with tumor progression; therefore, the platform synergizes with PD-1 blockade to achieve antitumor therapeutic effects 192. Collectively, these nanoplatforms primarily enhance antitumor efficacy by activating mitophagy.

Conversely, nanoplatforms can amplify mitochondrial injury via mitophagy inhibition. Deng et al. combined biomimetic RH-NPs, 4T1/mitochondrial membrane-cloaked Ca@GOx, with chloroquine nanoparticles (CQ-NPs) to induce calcium overload, ROS burst, and gasdermin E (GSDME)-mediated pyroptosis, and block compensatory mitophagy to boost CD8^+^ T-cell infiltration in breast cancer 193. Gong et al. coupled RGD-targeted MIL-100(Fe), a mesoporous silica nanoparticle, with Mdivi-1 to convert hydrogen peroxide (H_2_O_2_) to hydroxyl radicals (•OH) and block mitochondrial clearance, suppressing endometrial cancer growth 194. Zhuang et al. synthesized a near-infrared-II (NIR-II) type I photosensitizer (MTC) to induce lipid peroxidation, ferroptosis, and pyroptosis, while stalling the autophagosome flux to amplify immunogenic responses in tumor cells 195. Qiu et al. developed pH-sensitive Res@ZIF-90 to deliver resveratrol to tumor mitochondria, where resveratrol downregulates mitochondrial biogenesis markers (PGC-1α, TFAM), fusion markers (MFN1, OPA1), and mitophagy (PINK1) markers, while upregulating fission markers (Drp1, FIS1). The modulation collapses ΔΨm and enhances cytotoxicity 196. Zheng et al. assembled a Z-scheme BP-M-PtCu3 nanocomposite for ultrasound-activated sonodynamic therapy. The nanocomposite boosts ROS production, releases copper ions (Cu^2+^) to raise the lysosomal pH, blocks the mitophagic flux, and eradicates TNBC under photoacoustic (PA)/computed tomography (CT) guidance 197. Taken together, these mitophagy-modulating platforms exhibit potent antitumor efficacy, providing a versatile paradigm to enhance precision and efficacy of cancer therapy.

#### 3.4.4. Analysis of nanocarrier systems

Different nanocarriers for mitophagy-mediated antitumor therapy exhibit distinctly different characteristics and performances. The key differences among these platforms lie in targeting accuracy, responsiveness to the TME, drug loading capacity, and biocompatibility. The advantages and limitations of most commonly used nanocarriers for mitophagy modulation agents are compared and they are shown in Table 2.

Polymeric nanocarriers offer unparalleled versatility in delivering agents for modulating mitophagy. The structural plasticity of polymeric nanocarriers allows for fine-tuning the biodegradation rate of the nanocarriers *in vivo* by controlling the ratio and molecular weight of the monomers, thus ensuring controlled drug release at the tumor site and protecting the mitochondria of healthy tissues 200-202. Furthermore, their superior co-delivery architecture enables the simultaneous integration of mitophagy regulators, chemotherapeutic drugs, and immune adjuvants, achieving highly efficient synergistic therapy against tumor cells 203, 204. The inherent adaptability of the polymer nanocarrier surface also allows for the conjugation of mitochondria-targeting groups, such as TPP or MITO-Porter, and tumor-specific responsive molecules, such as MMP-2 sensitive peptides. These characteristics of polymer nanocarriers can be exploited to significantly enhance the specificity for tumor mitochondria and minimize off-target accumulation 205, 206.

#### 3.4.5. Strategies to enhance safety for nanocarrier systems

Mitochondria, a core organelle in high-energy-consuming tissues such as the heart and brain, are crucial for maintaining normal physiological activities. Traditional small-molecule mitophagy inhibitors cannot distinguish mitochondria between tumor tissues and normal tissues due to a lack of targeting specificity, leading to severe systemic toxicity and off-target damage to high-energy-consuming organs. In contrast, nanocarrier systems, with their unique structural and functional advantages, could achieve a balance between efficacy and safety through precise targeting strategies 207, 208.

By harnessing mitochondria-specific ligands, the abnormal mitochondrial membrane potential or highly expressed membrane proteins of tumor cells, nanocarrier systems can be surface-engineered to significantly improve their accumulation efficiency at the lesion site and reduce their non-specific binding to mitochondria in normal tissues 209. For example, TPP-modified polymer nanocarriers can precisely target tumor mitochondrial membranes through electrostatic interaction to achieve targeted drug delivery 210

Nanocarriers can also be engineered with TME responsiveness by introducing GSH-sensitive disulfide bonds, pH-sensitive hydrazone bonds, or enzyme-sensitive peptide bonds to ensure that mitophagy modulators from nanocarriers are released locally at the lesion site. This design can effectively avoid drug leakage in the circulatory system and accumulation in normal tissues. For example, high-concentration-glutathione-responsive PLGA nanocarriers degrade rapidly after entering tumor cells, however, they can maintain their structural stability in a low-concentration-glutathione environment of normal cells, thus markedly reducing their systemic risks 211, 212.

In addition, the feasibility of DDS to improve safety profile could be realized using a “low-dose sustained release” design concept. The slow degradation features of nanocarriers endow the carriers with an inability of instantly releasing drugs from them to organs such as heart and liver during circulation, which in turn lowers acute toxicity 213, 214. For instance, injectable hydrogel nanosystem could construct the persistent drug depots in tumor site for sustained releasing of mitotophagy regulators. This strategy allows for mitotophagy modulation with therapeutic efficacy and improves mitotophagy modulators by dulled administration frequency and minimized off-target mitochondrial impairments 215, 216.

## 4. Conclusion and Perspectives

### 4.1. Challenges in Mechanistic Research

Although great progress has been made, many challenges are still needed to be address in deciphering the regulatory role of mitophagy in tumors. The mitophagy pathway is highly spatiotemporal dependent, yet its intertissue/interpathological-stage dependence on the PINK1-Parkin pathway, receptor-mediated pathways and MDV-mediated pathways has been less systematically comprehended. The differences in the characteristics of different populations can be revealed with single-cell multi-omics technology, providing a theoretical basis for accurate targeted therapy.

**Figure 9:**
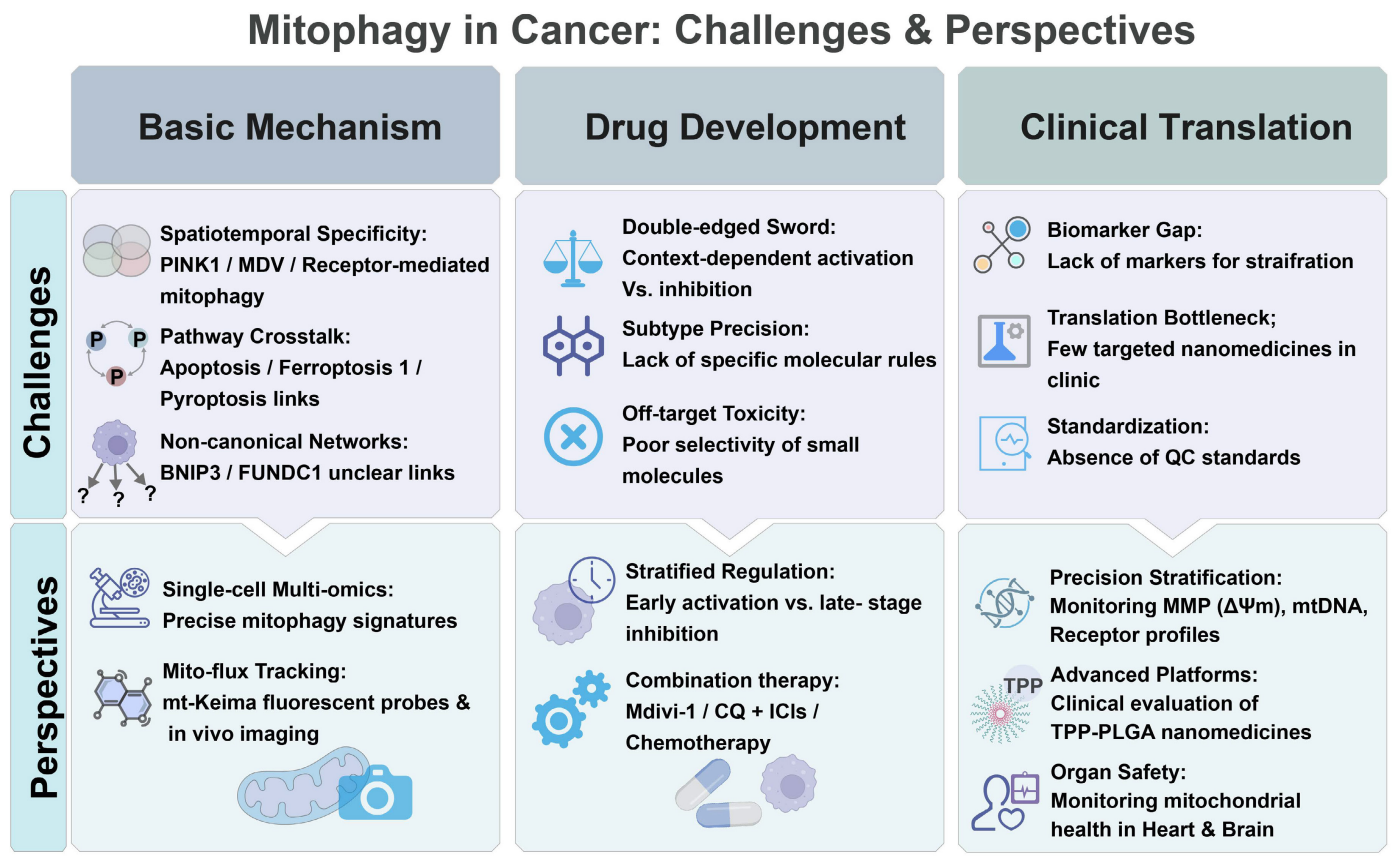
Illustration of challenges and perspectives of mitophagy in cancer. Created in https://BioRender.com

The interaction mechanisms between mitophagy and other cell death pathways (such as apoptosis, ferroptosis, or pyroptosis 217) are very complicated. Conventional detection methods have difficulty in distinguishing selective mitophagy from general autophagy effectively. Hence, it is important to further establish novel specific detection techniques, such as the mt-Keima fluorescent probe and *in vivo* autophagy flux imaging technique for specific investigation towards mitophagy in combination therapy. In addition, the noncanonical axis in BNIP3/NIX and FUNDC1 regulatory network has not been fully demonstrated, and its roles in tumor-specific metabolic reprogramming and immune microenvironment modulation needs to be further investigated (**Figure 9**).

### 4.2. Therapeutic Targeting Directions

Given the “double-edged sword” effect of mitophagy, precise stratified regulation is a promising future research direction. Treatment strategies should be dynamically adjusted based on the tumor stage, molecular subtype, and microenvironment characteristics.

**Mitophagy inhibition:** Mitophagy inhibition strategies are applicable for advanced tumors that rely on autophagy to develop chemoresistance or immune resistance, and malignant tumors with a high expression level of PINK1/Parkin/FUNDC1, such as cisplatin-resistant ovarian cancer and glioblastoma. By inhibiting mitophagy, metabolic adaptation and immune evasion of tumor cells can be blocked, significantly enhancing their sensitivity to chemotherapy. For example, the combination of Mdivi-1 and PD-1 inhibitors has shown a significant improvement in the objective response rate of advanced non-small cell lung cancer.

**Mitophagy activation:** Mitophagy activation strategies can be implemented to block early carcinogenesis by clearing damaged mitochondria, and they are applicable for metabolic liver cancer or oxidative stress-sensitive breast cancer. Furthermore, excessive mitophagy activation is an effective approach to disrupting the energy metabolism of drug-resistant tumors. Studies have shown that soy isoflavones can inhibit occurrence and metastasis of osteosarcoma by activating mitophagy, with low toxicity to normal tissues.

**Optimization of combination therapy:** Mitophagy modulators may be applied as a sensitizer in combination therapy. Combination of mitophagy inhibitors with ICIs should be explored to reverse immune resistance, or combination of mitophagy activators with chemotherapy drugs to amplify oxidative damage. In addition, combining modulators with metabolic inhibitors to block metabolic reprogramming is very promising. For example, the combination of chloroquine and PI3K/AKT inhibitors has shown efficacy in reversing drug resistance in triple-negative breast cancer treatment.

### 4.3. Clinical Translation Pathway

Clinical translation of mitophagy-based therapy faces several key bottlenecks. Identification and validation of specific biomarkers—including mitochondrial membrane potentials, ubiquitinated protein levels, receptor expression profiles, and released mtDNA—are crucial for patient stratification and dynamic monitoring of treatment efficacy.

Currently, there is no mitochondria-targeting nanomedicine in active clinical trials; therefore, there is a pressing need to accelerate clinical evaluation of nanoplatforms with well-defined mechanisms and great biocompatibility (e.g., TPP-modified PLGA carriers) for mitophagy modulators. Quality control standards for nanoplatforms encompassing particle size distribution, targeting efficiency, and release kinetics should be established to ensure their safety for clinical application. Clinical research designs should adhere to the "mechanism-oriented" principle, and treatment plans are tailored to mitophagy characteristics of patients. For example, a combination of mitophagy inhibitors and PD-L1 inhibitors could be used for advanced lung cancer patients with activated PINK1-Parkin pathways; while a combination of siRNA and paclitaxel delivered by a nanocarrier could be applied for drug-resistant breast cancer patients with a high FUNDC1 expression level. Throughout the entire translation process, the mitochondrial function in high-energy-consuming tissues, such as the heart and brain, must be closely monitored to ensure long-term safety of this cutting-edge therapy.

In conclusion, mitophagy, as a potential target for cancer treatment, relies on a deep understanding of its mechanisms, innovative design of targeting strategies, and systematic advancement of clinical translation for precise regulation. With the integrated development of multidisciplinary technologies, targeted therapy of mitophagy is expected to become an important means to overcoming drug resistance and improving clinical efficacy, thus providing personalized treatment options for cancer patients.

## Figures and Tables

**Table 1:** Small molecules that affect mitophagy in various cancers

Classification	Names	Mechanisms	Cancer types	References
Mitophagy inhibitor	Tanshinone I	Inhibition of mitophagy through inhibiting the expression of PINK1	Cervical cancer	26
	S1g-2	Disrupting Hsp70-Bim protein‒protein interaction to selectively inhibit stress-induced mitophagy	Chronic myeloid leukemia	151
	DC-ATG4in	Directly binding to ATG4B and inhibiting its enzyme activity to block mitophagy	HCC	155
	Mdivi-1	Inhibiting DRP1 to reduce mitophagy and increasing cell apoptosis	Ovarian cancer	165
	Fluorizoline	Pro-apoptotic PHB-binding to block the process of mitophagy	Cervical carcinoma, lung cancer	166
	Rapamycin	Promoting mitophagy via activation of the miR-26a-5p/DAPK1 pathway	Glioma	156
	Rhopaloic acid A	Triggering mitochondria damage-induced apoptosis by JNK/BNIP3/Nix-mediated mitophagy	Oral squamous cell carcinoma	157
	Morusin	Binding to the active domain of ATP-citrate lyase (ACLY), leading to ROS accumulation and activation of PINK1/Parkin-mediated mitophagy	HCC	158
	PMI	Stabilizing Nrf2 and increasing the P62 level to drive mitophagy	Neuroblastoma	159
Mitophagy inducer	Salinomycin	Inducing ROS accumulation and mitochondrial membrane depolarization	Prostate cancer	160
	Ginsenoside	Activating the PINK1-Parkin signaling pathway and recruiting Parkin and Ub to mitochondria to induce mitophagy	Colon cancer	161
	Dihydroergotamine tartrate	Decreasing membrane permeability, increasing ROS generation, disturbing ATP production, and finally inducing cell death by mitophagy and apoptosis	Lung cancer	162
	Curcumin	Initiating mitophagy with ultrasound treatment	Nasopharyngeal carcinoma	163
	1,10-Phenanthroline	Disruption of mitochondrial dynamics and promotion of mitochondrial dysfunctions to provoke mitophagy	Cervical carcinoma	164
	Soy isoflavone	Blocking the AKT/mTOR signaling pathway to induce mitophagy	Osteosarcoma	152

Footnote: Abbreviations: ACLY (ATP citrate lyase), ATG4B (autophagy-related protein 4B), DRP1 (dynamin-related protein 1), Hsp70 (heat shock protein 70), JNK (c-Jun N-terminal kinase), PINK1 (PTEN-induced putative kinase 1), ROS (reactive oxygen species), mTOR (mammalian target of rapamycin), DAPK1 (death-associated protein kinase 1). All mechanisms are based on preclinical studies in cell lines or animal models; references correspond to the original research reporting each compound's mitophagy-modulating activity.

**Table 2:** A comparative overview of nanocarrier performances specifically within the framework of mitophagy-modulated cancer therapy.

Nanocarrier Type	Advantages	Limitations	Typical Application
Liposomes	Exceptional biocompatibility and membrane fusion facilitate efficient penetration through cellular and mitochondrial membranes; established manufacturing processes allow for large-scale production.	Poor stability within the TME often leads to premature drug leakage; targeting specificity relies heavily on surface modifications and the specificity is sensitive to fluctuations in the mitochondrial membrane potential.	Treatments requiring rapid onset and minimal toxicity, such as combination chemotherapy for lung or breast cancer 198.
Polymers	Robust responsiveness to microenvironmental stimuli, a superior co-delivery capacity, and highly flexible surface functionalization enable precise targeting through conjugated peptides or antibodies and controlled released within the TME 199.	Slow biodegradation *in vivo* may raise long-term safety concerns; stringent quality control should be implemented to reduce the risk of residual organic solvents during synthesis.	Synergistic therapy for drug-resistant malignancies, including co-delivery of mitophagy regulators and chemotherapeutics for pancreatic or liver cancer.
Hybrid nanosystems	Multiple carrier types are synergistically integrated to facilitate sophisticated multimodal therapies.	Complex synthesis protocols challenge batch-to-batch consistency; poorly defined *in vivo* metabolic pathways require extensive long-term safety validation.	Precision combination therapy for advanced solid tumors, such as photothermal-immune-mitophagy synergistic treatment for glioblastoma.

## Abbreviations

ATG: Autophagy-related protein
BCL2L13: BCL2-like 13
BNIP3: BCL2-interacting protein 3
cGAS-STING: Cyclic GMP-AMP synthase-stimulator of interferon genes
DAMPs: Damage-associated molecular patterns
DRP1: Dynamin-related protein 1
FUNDC1: FUN14 domain-containing 1
ICD: Immunogenic cell death
LC3: Light chain 3
LIR: LC3-interacting region
MDV: Mitochondria-derived vesicle
mtDNA: Mitochondrial DNA
OMM: Outer mitochondrial membrane
OPTN: Optineurin
PARK2: Parkin gene
PARK6: PINK1 gene
PD-L1: Programmed death-ligand 1
PLGA: Poly(lactic-co-glycolic acid)
PINK1: PTEN-induced putative kinase 1
ROS: Reactive oxygen species
SQSTM1/p62: Sequestosome-1
TPP: Triphenylphosphine
TME: Tumor microenvironment
ULK: Unc-51-like autophagy-activating kinase
UBD: Ubiquitin-binding domain
WIPI: WD-repeat protein interacting with phosphoinositides
